# The exchange of the fast substrate water in the S_2_ state of photosystem II is limited by diffusion of bulk water through channels – implications for the water oxidation mechanism†

**DOI:** 10.1039/d1sc02265b

**Published:** 2021-09-01

**Authors:** Casper de Lichtenberg, Christopher J. Kim, Petko Chernev, Richard J. Debus, Johannes Messinger

**Affiliations:** Department of Chemistry, Umeå University Linnaeus väg 6 (KBC huset), SE-901 87 Umeå Sweden johannes.messinger@kemi.uu.se; Molecular Biomimetics, Department of Chemistry – Ångström Laboratory, Uppsala University POB 523 SE-75120 Uppsala Sweden johannes.messinger@kemi.uu.se; Department of Biochemistry, University of California Riverside California 92521 USA richard.debus@ucr.edu

## Abstract

The molecular oxygen we breathe is produced from water-derived oxygen species bound to the Mn_4_CaO_5_ cluster in photosystem II (PSII). Present research points to the central oxo-bridge O5 as the ‘slow exchanging substrate water (W_s_)’, while, in the S_2_ state, the terminal water ligands W2 and W3 are both discussed as the ‘fast exchanging substrate water (W_f_)’. A critical point for the assignment of W_f_ is whether or not its exchange with bulk water is limited by barriers in the channels leading to the Mn_4_CaO_5_ cluster. In this study, we measured the rates of H_2_^16^O/H_2_^18^O substrate water exchange in the S_2_ and S_3_ states of PSII core complexes from wild-type (WT) *Synechocystis* sp. PCC 6803, and from two mutants, D1-D61A and D1-E189Q, that are expected to alter water access *via* the Cl1/O4 channels and the O1 channel, respectively. We found that the exchange rates of W_f_ and W_s_ were unaffected by the E189Q mutation (O1 channel), but strongly perturbed by the D61A mutation (Cl1/O4 channel). It is concluded that all channels have restrictions limiting the isotopic equilibration of the inner water pool near the Mn_4_CaO_5_ cluster, and that D61 participates in one such barrier. In the D61A mutant this barrier is lowered so that W_f_ exchange occurs more rapidly. This finding removes the main argument against Ca-bound W3 as fast substrate water in the S_2_ state, namely the indifference of the rate of W_f_ exchange towards Ca/Sr substitution.

## Abstract 2

Access to the oxygen-evolving complex in photosynthesis is restricted by specific barriers in the channels connecting the Mn_4_CaO_5_ catalyst with bulk water. Together with other recent data, this finding allows assigning the two substrate waters.

## Graphical abstract

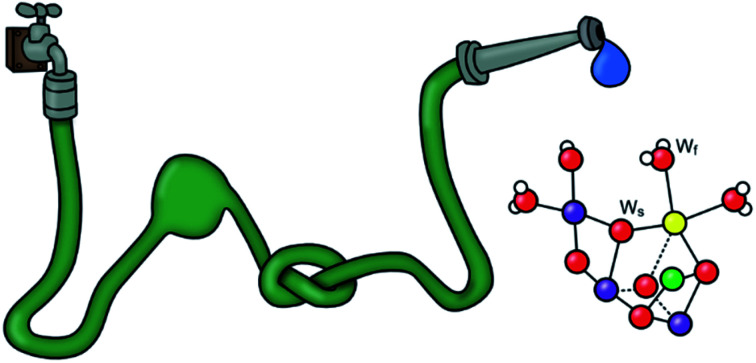

## Introduction

Photosynthesis performed by plants, algae and cyanobacteria is critical for life on Earth as it releases molecular oxygen into the atmosphere and stores solar energy as biomass. Utilizing sunlight, the protein complex photosystem II (PSII) generates and stabilizes charge pairs that are employed for the extraction of 4 electrons and 4 protons from 2 water molecules, and to reduce plastoquinone to plastohydroquinone.^1,2^ The solar-to-chemical energy conversion efficiency of PSII has been estimated to reach values of up to 15%.^3^

The water oxidation reaction is catalyzed by a metal–oxygen cluster comprising the metals manganese and calcium in a 4 : 1 stoichiometry as well as five oxo bridges (O1–O5).^4–6^ During the reaction cycle, the Mn_4_CaO_5_ cluster is stepwise oxidized by light-induced charge separations in the chlorophyll containing reaction center of PSII. Thereby, it attains four discrete reaction intermediates (S_0_–S_3_) and one highly reactive transient (S_4_).^7–10^ The S_1_ state is dark-stable, and the S_2_ → S_3_ transition involves the association of a new water molecule (W_N1_), yielding a Mn_4_CaO_6_ cluster as the last stable intermediate before O_2_ formation.^11–17^ The next light-induced charge separation triggers the S_3_ → S_4_ → S_0_ transition, which not only involves the O–O bond formation, but also O_2_ release and the concomitant filling of the open coordination site by one of the terminal water ligands (W3 or W2) as well as the binding of a new water molecule (W_N2_).^9,12,18,19^ All S state transitions, with the exception of S_1_ → S_2_, are coupled to proton release into the bulk, keeping the total charge of the cluster at 0 or +1, respectively.^20^ Proton release is facilitated by an intricate H-bonding network that is pivotal to the function of PSII and its earth-abundant water oxidation catalyst.^17,21–24^

**Fig. 1:**
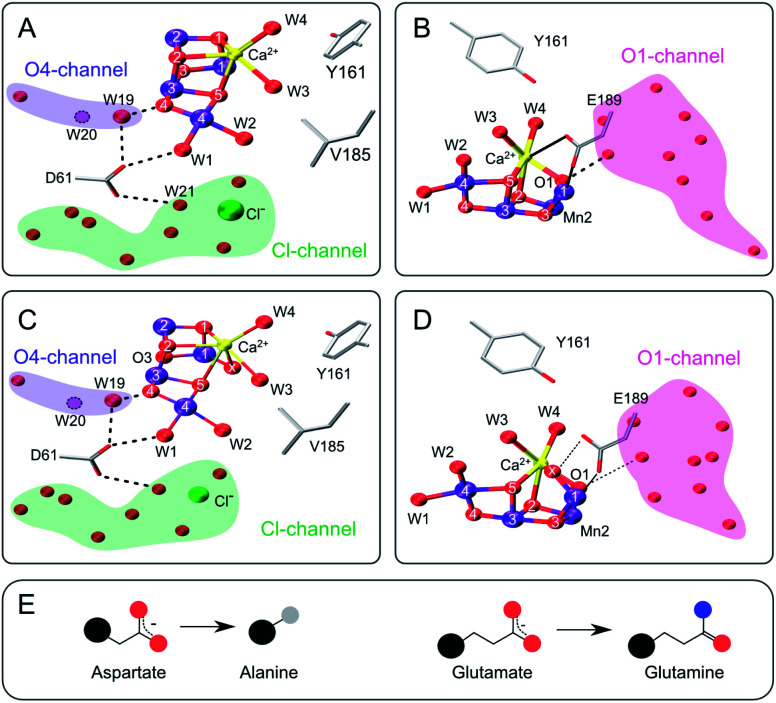
Structure of the Mn_4_CaO_5_ cluster with selected ligands and water molecules in the S_2_ (panels A and B) and S_3_ (C and D) states of photosystem II (PDB: 6DHF & 6DHO). Note that the cluster has a sixth oxygen bridge labelled X in the S_3_ state. Panels A & C highlight the position of D61 and panels B & D that of E189 in relation to the Mn_4_CaO_5/6_-cluster and the O4 (blue), Cl1 (green) and O1 (pink) water/proton channels. Potential hydrogen bonds are shown as dashed lines, while the coordination of E189 to Ca and Mn is indicated with solid lines. The position of W20, which is not resolved in the S_2_- and S_3_-state structures, is indicated by a dashed circle. E: Cartoon of the D to A mutation (left) and the E to Q mutation (right). Color code: large black sphere – peptide backbone; red - oxygen; blue - nitrogen; purple – manganese; yellow – calcium; green – chloride; grey - methyl group. The molecular representations were generated with VMD.^102^

The Mn_4_CaO_5_ cluster is frequently described as having a ‘chair’-like structure, with the base formed by a Mn_3_CaO_4_ hetero-cubane and the back by the fourth Mn ion (Mn4) that is connected to the base *via* the oxygen bridges O5 and O4 (Fig. 1).^6^ As there is no bond between O5 and Mn1, the structure is referred to as ‘open cubane’.^5,15^ Importantly, this structure binds four water molecules, two at Mn4 (W1, W2) and two at Ca (W3, W4), while all other coordination sites, except one at Mn1, are filled by five oxo-bridges, six bridging carboxylates and one histidine ligand.^6,15^ In the S_0_ state, the four Mn ions have the oxidation states Mn_4_(III,IV,III,III) (oxidation states given in the order Mn1 through Mn4), and up to S_3_ all transitions involve a Mn(iii) → Mn(iv) oxidation (for review see ref. 25, 26), although for the S_3_ state also a small equilibrium concentration of a peroxidic intermediate has been proposed to exist.^1,21,27,28^ By contrast, the S_4_ state likely involves oxygen radical formation.^12^ Alternatively, electronic compositions of Mn(IV,IV,IV,V), Mn(III,III,IV,VII), or superoxo intermediates have been proposed for S_4_ (for review see ref. 29, 30 and ESI Fig. S1†).

**Scheme 1:**
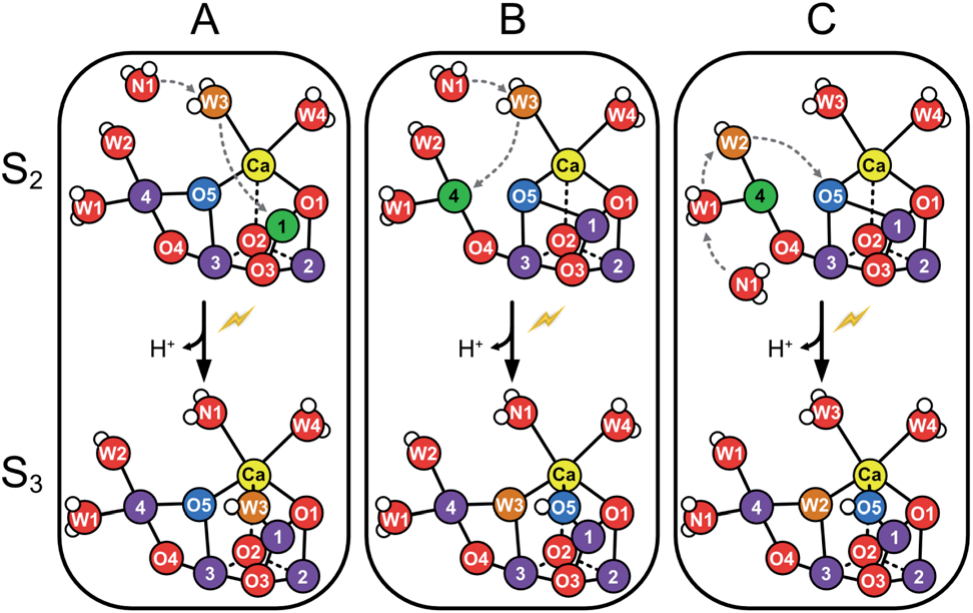
Suggested routes for insertion of W_N1_ and formation of the Ox hydroxo bridge during the S_2_ → S_3_ transition. Panels A and B show two proposed pathways for W3 insertion. Pathway A starts from the more stable, open cube (S_2_^A^) conformation of the Mn_4_CaO_5_-cluster. W3 is inserted into the Ox site between Ca and Mn1, while W_N1_ replaces W3.^32,33^ B: The Mn_4_CaO_5_-cluster attains first the S_2_^B^ conformation before W3 binds to Mn4. W3 then flips into the O5 binding site, while O5 moves into the Ox position and W_N1_ replenishes the original W3 coordination site at Ca.^32,33^ C: The pivot or carousel mechanism requires also that the cluster attains first the less stable S_2_^B^ conformation. Binding of W_N1_ to the five-coordinate Mn4(III) induces a cascade of water/oxygen relocations allowing W1 to replace W2, W2 to flip into the O5 position, and O5 to occupy the Ox site.^36,37^

**Scheme 2:**
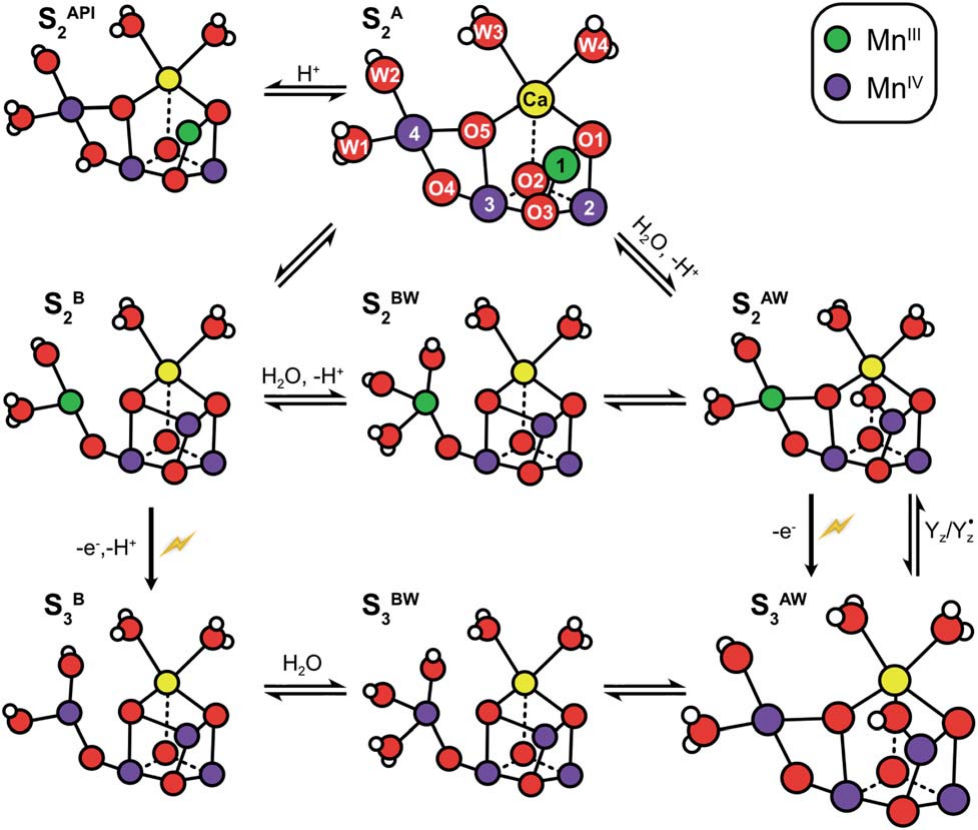
Structural flexibility of the Mn_4_CaO_5/6_ cluster in photosystem II. S_2_^A^ and S_3_^AW^ are the most stable structures of the S_2_ and S_3_ states and have been observed by crystallography at room temperature.^14,15,17^ S_2_^B^, S_2_^BW^, S_2_^AW^ and S_2_^API^ are computational structures that were proposed to give rise to the S_2_^HS^ EPR signal, and or have been suggested as intermediates during O5 exchange against bulk water.^41,46,48,55^ S_3_^B^ and S_3_^BW^ have been supported by EPR spectra obtained when S_2_ samples were advanced to S_3_ under conditions that may block water insertion, and have been suggested as intermediates during the S_2_ → S_3_ transition.^36,92^ They are also involved in substrate water exchange.^44,46,55^ For example, in the S_2_^A^ state O5 (W_s_) exchanges *via* the S_2_^AW^, S_2_^BW^ and S_2_^B^ states, while its exchange in the S_3_^AW^ state requires the equilibrium of S_3_^AW^ Y_Z_ with S_2_^AW^ Y_Z_˙ or alternatively the transition into the S_3_^BW^ and S_3_^B^ states. Color code: Mn^IV^ purple, Mn^III^ green, Ca yellow, O red, H white. The flash indicates a light-induced charge separation in PSII.

The structure of the Mn_4_CaO_5_ cluster is flexible. In the S_2_ → S_3_ transition it takes up one additional water molecule (W_N1_) and a new hydroxo/oxo bridge (Ox/O6; here after Ox) is formed between Ca and Mn1.^14,15,17,21,31^ The precise mechanism for this is under debate and the three discussed options are depicted in Scheme 1.^32–37^ In addition to this water uptake (denoted by a W superscript; Scheme 2), in each S state the cluster can attain at least two different conformations.^18,38–41,56^ This is best documented for the S_2_ state, where the two conformations give rise to the low spin (LS) S_2_*g* = 2 multiline and the broad high-spin (HS) *g* = 4–6 EPR signals, respectively. The well-characterized open cube (S_2_^A^) structure gives rise to the S_2_^LS^ signal, while for the S_2_^HS^ state several structures have been proposed: the closed cube S_2_^B^,^34,41^ the open cube water bound S_2_^AW^^42-46^ and the protonation isomer S_2_^API^^47,48^ (Scheme 2). Among these, the S_2_^B^ and the S_2_^API^ structures provide the best computational explanation for the *g* = 4 EPR signal, while the S_2_^AW^ state, which has a structure akin to the S_3_^AW^ state, is favored on the basis of substrate water exchange experiments, and because it provides a straightforward explanation for the low transition temperature of S_2_^HS^ to S_3_^AW^.^44–46,49^ For all S states, the S^A^ structures dominate under most conditions, except for the S_3_ state, where S_3_^AW^ is most stable.^19,29^

Identification of the two substrate water binding sites in the four discrete intermediates of the reaction cycle would provide a solid basis for decoding the mechanism of biological water oxidation. While there are several ways to identify water molecules bound to or near the Mn_4_CaO_5/6_ cluster, only the determination of the isotopic composition of the O_2_ produced after a rapid enrichment of the sample with H_2_^18^O by membrane inlet mass spectrometry (MIMS) allows obtaining a unique experimental signature for the two substrates: their exchange rates with bulk water.^18,50,51^

Using this approach, it was shown that the two substrates are bound differently in the S_2_ and S_3_ states.^18,52^ The faster exchanging substrate water is referred to as W_f_, while the slower one is denoted as W_s_. For the S_0_ and S_1_ states, only the exchange rates of W_s_ were determined. However, since no water binding events are known for the S_0_ → S_1_ and S_1_ → S_2_ transitions, both substrates must be bound already also in these early S states.^46^ Connecting the water exchange data with emerging structural and spectroscopic information led to the proposal that W_s_ is the central μ_3_-oxo bridge, today known as O5.^9^ This was subsequently supported by theoretical and spectroscopic^12,53,54^ as well as further MIMS studies.^18^ Exchange of O5 has been shown to be a multistep process in which O5 is brought into a terminal position on Mn4 where it is fully protonated. In this process, the Mn_4_CaO_5/6_ cluster attains several of the alternative conformations shown in Scheme 2.^44,46,55^ For example, O5 (Ws) would exchange in the S_2_^A^ state by first forming S_2_^AW^ through the uptake of one water, and then changing conformation to a S_2_^BW^ state, which *via* equilibrium with the S_2_^B^ state allows exchange of O5 with bulk water.^44,46,55^

By contrast, the assignment of W_f_ is controversial. FTIR and snapshot crystallographic studies as well as a number of DFT calculations suggest that W_f_ is bound as W3 to Ca in the S_2_ state, but then forms a bridge between Ca and Mn upon S_3_ state formation *via* insertion pathways A or B (Scheme 1).^14–17,21,33^

On the other hand, present MIMS experiments favor the terminal water ligand W2 as W_f_, because the exchange of W_f_ is firstly several orders of magnitude slower than would be expected for a terminal water ligand on Ca,^9,18,57^ and secondly independent of Ca/Sr-substitution in both the S_2_ ^46^ and S_3_ ^18,58,59^ states. In addition, W_f_ exchange becomes observable first in the S_2_ state, and then slows upon S_3_ and S_3_Y_Z_˙ state formation, making a diffusion limitation that could obscure the Ca/Sr dependence seemingly unlikely. By contrast, these two observations can be well explained with W2 as W_f_ by the known oxidation of Mn4 during the S_1_ → S_2_ transition and the need to involve electron back donation of Y_Z_ for W_f_ exchange in the S_3_ state.^9,18,59^ Absence of a diffusion limitation is apparently further supported by molecular dynamics (MD) calculations that predict water access in the 50 ns to 100 μs time range,^60,61^*i.e.* orders of magnitude faster than W_f_ exchange (50–100 ms).^44,46,57^

Three channels have been identified that lead to the Mn_4_CaO_5_ cluster: the O1 or ‘large’ channel, the O4 or ‘narrow’ channel, and the Cl1 or ‘broad’ channel (Fig. 1). While the O1 and Cl1 channels both split into two branches (A, B),^15,17^ all three channels have been variously proposed to be involved in either proton, dioxygen and/or water transport during various S state transitions, for review see.^17,21,60,62,63^ Recent room temperature and cryogenic X-ray crystallography studies favor that water access to the catalytic site occurs *via* the O1 channel as it shows the largest variation in water positions between studies and S states.^15,17,64^ By contrast, previous theoretical studies suggested that water is delivered through the O4 channel to the Mn4 site and is inserted during the S_2_ → S_3_ transition *via* the pivot/carousel mechanism (Scheme 1C).^36,37^ Recent mass spectrometric studies analyzing the oxidative damage to the D1, D2 and CP47 proteins caused by the formation of reactive oxygen species (ROS) at the Mn_4_CaO_5/6_ cluster under illumination support both the B branch of the Cl1 channel and the O1 channel as water access pathways.^63,65,66^

To probe if the fast water exchange (W_f_) in the S_2_ state is limited by diffusion through channels or by the chemical exchange process, we study here the effects of the D1-D61A and D1-E189Q mutations on the rates of substrate water exchange with bulk water in the S_2_ and S_3_ states.

The D61 residue is located close to Mn4 at the apex between the potential O4 and Cl1 substrate channels (Fig. 1). D61 hydrogen bonds W1 and some further waters in its surroundings. If this aspartate (D) residue is mutated to either asparagine (N) or alanine (A), O_2_ production decreases by ∼75–80%, and the S_1_ → S_2_ and S_2_ → S_3_ transitions are decelerated by factors of 2–3.^67^ Meanwhile, O_2_ release in the S_3_ → S_0_ transition is retarded 20–30 fold.^67–69^ These functional effects were attributed to poor proton abstraction from the mutants, identifying this residue as an important proton relay.^68,70,71^ It may be speculated that if W2 were a substrate, its exchange would be greatly affected by the D61A mutation. The S_3_ state exchange rates were previously measured for the D61N mutant, showing 6-fold and 3-fold slower exchange rates for W_f_ and W_s_, respectively.^72^

E189 is located at the end of the O1 channel. In the S_1_ and S_2_ states, E189 is a ligand of Mn1, and it also weakly ligates Ca. Recently it was shown, by time-resolved X-ray crystallography, that during the S_2_ → S_3_ transition E189 detaches from Ca before Ox is inserted, and afterwards hydrogen bonds Ox (Fig. 1B and D).^15,17,21^ Consequently, this glutamate residue (E189) may be important for the insertion of Ox during the S_2_ → S_3_ transition, the exchange of Ox by bulk water in the S_3_ state, and O–O bond formation. Only a handful mutations of E189 yield active PSII centers, namely isoleucine (I), lysine (K), leucine (L), glutamine (Q) and arginine (R).^73^ E189Q is a conservative mutant, as it is of similar size and retains the ability to act as bidentate ligand (Fig. 1E). While the S_2_^LS^ signal is not perturbed by the mutation, the oxygen evolution activity is decreased by ∼30%,^73^ indicating that some transition in the catalytic cycle does not function optimally. For the S_3_ state, an up to 2-fold faster substrate water exchange was reported previously.^74^

## Experimental procedures

### Preparation of photosystem II core complexes

*Synechocystis* sp. PCC 6803 strains, with a 6xHis-tag fused to the CP47 gene, expressing the psbA2-gene (WT, D1-D61A or D1-E189Q) were propagated in BG11 medium supplemented with glucose in glass carboys and grown as previously described.^70^ Thylakoid membranes and core complexes were prepared as described previously.^70^ The PSII core complexes were suspended in 1.2 M betaine, 10% (v/v) glycerol, 50 mM MES-NaOH (pH 6.0), 20 mM CaCl_2_, 5 mM MgCl_2_, 50 mM histidine, 1 mM EDTA, and 0.03% (w/v) n-dodecyl β-D-maltoside, and were concentrated to ∼1 mg of Chl mL^−1^. The samples were then divided into 100 μL aliquots and flash-frozen in liquid N_2_. Finally, samples were stored at −80 °C.

### Time-resolved membrane-inlet mass spectrometry

Substrate–water exchange rates were measured at 10 °C employing an isotope ratio mass spectrometer (Finnigan Delta Plus XP) featuring 7 Faraday cups (*m*/*z* 32, 34, 36, 40, 44, 46 & 48) and a 165 μL rapid mixing reaction cell that was connected to the spectrometer through a stainless steel pipe that passed through a Dewar filled with liquid N_2_.^51^ After thawing, the PSII core complexes were washed (total dilution factor: 100–1000) in 50 mM MES-NaOH pH/pD 6.5, 1 M betaine, 15 mM CaCl_2_, 15 mM MgCl_2_ using an Amicon Ultra-0.5 centrifugal filter unit and finally concentrated to 0.15–0.2 mg Chl per mL. After a saturating preflash (5 μs FWHM), the sample was dark-adapted for 1 hour at room temperature. Prior to loading in dim green light, 0.3 mM (final concentration) 2,6-dichloro-1,4-benzoquinone was added.

A modified gas-tight syringe (Hamilton CR-700-50) with an air pressure driven, computer triggered piston, previously loaded under N_2_ atmosphere with ∼22 μL 97% H_2_^18^O, was employed for rapid (∼6 ms) isotope enrichment to a final level of ∼12%.^57^

Residual O_2_ in the H_2_^18^O was estimated and removed from the data as described previously.^44^ The measurement sequences for all samples and S states are shown in ESI Fig. S2.† The substrate exchange rates (*k*_f1_, *k*_f2_, *k*_s1_ and *k*_s2_) for the fast and slow substrate waters were determined by a simultaneous fit of the *m*/*z* 34 and the *m*/*z* 36 data (for details see ESI Text 1 and Table S1†).

## Results

**Fig. 2:**
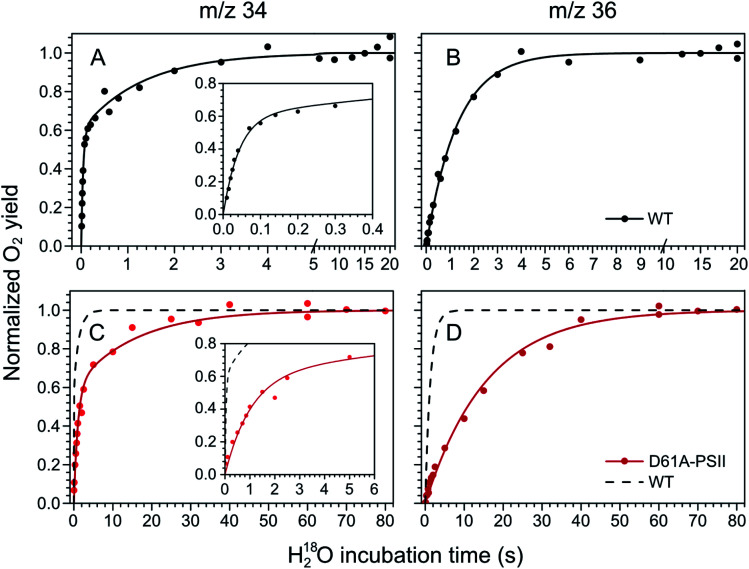
Substrate water exchange measurements in the S_3_ state of WT- (black) and D61A- (red) PSII core complexes of Synechocystis sp. PCC6803. The normalized oxygen yield of a flash given after different incubation times with H_2_^18^O in the S_3_ state are plotted. A and C show the results for single labelled oxygen (*m*/*z* 34), while panels B and D those for double labelled oxygen (*m*/*z* 36). Dots represent individual measurements, while solid lines the results of kinetic fits (Table 1). The fits of the WT-PSII substrate exchange are shown as a dashed line next to the D61A-PSII data for visual comparison. The inserts show an enlarged view of the fast exchange phase in the *m*/*z* 34 data. Observe differences in the time scales. The data were recorded at 10 °C, pH 6.5.

**Fig. 3:**
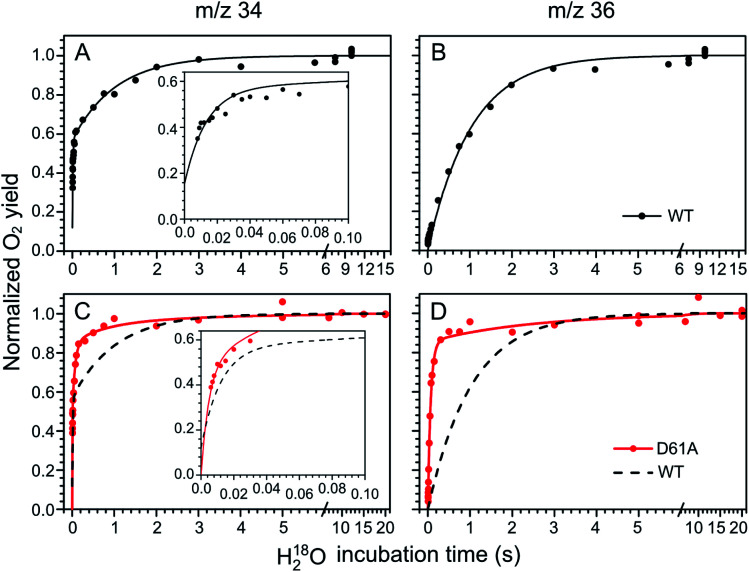
Substrate water exchange measurements in the S_2_ state of WT- (black) and D61A- (red) PSII core complexes of *Synechocystis* sp. PCC6803. The normalized oxygen yield of a double flash given after different incubation times with H_2_^18^O in the S_2_ state are plotted. A and C show the results for single labelled oxygen (*m*/*z* 34), while panels B and D those for double labelled oxygen (*m*/*z* 36). Dots represent individual measurements, while solid lines the results of kinetic fits (Table 1). The fits of the WT-PSII substrate exchange are shown as a dashed line next to the D61A-PSII data for visual comparison. The inserts show an enlarged view of the fast exchange phase in the *m*/*z* 34 data. Observe differences in the time scales. The data were recorded at 10 °C, pH 6.5.

The substrate water exchange rates of WT-, D61A- and E189Q-PSII core complexes from *Synechocystis* sp. PCC 6803 were studied in the S_2_ and S_3_ states of the oxygen-evolving complex at 10 °C, pH 6.5. For WT-PSII, the canonical biphasic exponential rise with a fast and slow phase^18^ was observed for the ^16,18^O_2_ signal from the *m*/*z* 34 cup in the S_2_ and S_3_ states (symbols in Fig. 2A and 3A). The biphasic rise shows that the two substrate waters are bound differently to the Mn_4_CaO_5_ cluster in these S states. Accordingly, they are referred to as the fast, W_f_, and slow, W_s_, exchanging substrate waters. The corresponding rates, *k*_f_ and *k*_s_, obtained from the kinetic fits (solid lines) are given in Table 1. For the ^18,18^O_2_ signal (*m*/*z* 36), which requires that both substrate waters exchange against H_2_^18^O added to the bulk water, a mono-exponential rise with the rate *k*_s_ was detected (Fig. 2B and 3B). This is expected, as this process is limited by the slower exchange process. The monophasic rise of the *m*/*z* 36 signal confirms that the two kinetic phases in the *m*/*z* 34 signal do not arise from sample heterogeneity.^51^

**Table 1:** Exchange rates of substrate water in the S_2_ and S_3_ states of photosystem II core complexes isolated from wild-type (WT), D1-D61A and D1-E189Q mutants of *Synechocystis* sp. PCC 6803. The rate constants and fractions of PSII centers were obtained from global fits of the ^16,18^O_2_ (*m*/*z* 34) and ^18,18^O_2_ (*m*/*z* 36) data displayed as lines in Fig. 2 and 3. The data were obtained at 10 °C and pH 6.5. For additional parameters see ESI Table S1

		WT-PSII		D61A-PSII				E189Q-PSII	
		*k*_f_	*k*_s_	*k*_f1_	*k*_s1_	*k*_f2_	*k*_s2_	*k*_f_	*k*_s_
S_3_	Fraction, %	100		100		0		100	
	Rate, s^−1^	23.4 ± 1.4	0.76 ± 0.03	0.97 ± 0.07	0.064 ± 0.003	—	—	24.6 ± 1.8	0.76 ± 0.04
	Mutant/WT	—	—	0.041 ± 0.004	0.084 ± 0.005	—	—	1.07 ± 0.08	1.00 ± 0.07
S_2_	Fraction, %	100		85		15		100	
	Rate, s^−1^	84 ± 5	0.97 ± 0.03	>300	15 ± 1	1.4 ± 0.9	0.4 ± 0.1	66 ± 5	0.94 ± 0.04
	Mutant/WT	—	—	>3.5	15 ± 1	0.017 ± 0.011	0.4 ± 0.1	0.79 ± 0.08	0.97 ± 0.05

In the S_3_ state, mutation of the D1–D61 residue to alanine led to a 24- and 12-fold slowing of W_f_ and W_s_ exchange (Fig. 2C and D, Table 1). This slowing is one of the largest effects of a mutation or biochemical change on substrate exchange kinetics observed thus far.^18^ For example, this change is 4-fold larger than the previously reported 6- and 3-fold decelerations for the D61N mutant.^72^ Notably, the monophasic rise of the *m*/*z* 36 signal was preserved (Fig. 2D).

In the S_2_ state, the same mutation had the opposite effect, *i.e.* a strong acceleration of the exchange was found for both substrates (Fig. 3C and D): 15-fold for W_s_ and more than 3.5-fold for W_f_, of which the rate could no longer be resolved with our present mixing system (Table 1).

However, detailed analysis showed that the exchange of both W_f_ and W_s_ were biphasic, and that in the smaller fraction, about 15%, the exchange of W_f_ and W_s_ occurred with rates that were slower than those of WT-PSII (Table 1). Thus, the *m*/*z* 34 data were fit with 4 kinetic phases instead of 2. This showed that in the S_2_ state of D61A-PSII two stable populations of the Mn_4_CaO_5_ cluster with possibly different substrates, exchange pathways or water accessibility must exist.

To probe the effects of H-bonding and of O–H bond breaking/formation on the exchange of substrate water in the S_2_ state of WT- and D61A-PSII, we performed the same experiments also in D_2_O (Fig. S3 and Table S2†). In general, the exchange rates of W_f_ and W_s_ were slower in D_2_O. W_f_ showed a corrected H/D isotope effect of ≤1.3. By contrast, W_s_ displayed an H/D isotope effect of 1.5 (WT) to 1.9 (D61A, larger fraction) and 2.8 (D61A, smaller fraction). In D61A-PSII, the smaller phase of W_f_ and W_s_ exchange increased from 15% (H_2_O) to 24% (D_2_O) (Table S2†).

Water exchange in the S_2_ and S_3_ states of the D1-E189Q mutant occurred with nearly identical rates as in WT-PSII. Only the exchange of W_f_ was retarded by ∼20% in the S_2_ state of the E189Q samples (Table 1; Fig. S4†). We note that a ∼2-fold acceleration was previously observed in the S_3_ state exchange rates of E189Q-PSII thylakoid membranes.^74^

## Discussion

In this study, we observed that the mutation of D61 to alanine had a strong effect on the exchange of both substrate waters in the S_2_ and S_3_ states, while the mutation of E189 to glutamine had essentially no influence on either W_f_ or W_s_ exchange. As D61 is close to W2, while E189 is near W3 and Ox, these results appear, at first glance, to favor W2 over W3 as fast exchanging substrate W_f_. However, because we previously showed that the substrate water exchange rates in PSII are strongly affected by conformational equilibria of the Mn_4_CaO_5/6_ cluster, and because the mutations are also located at the end points of water channels and may thereby affect the diffusion of water to the catalytic site, a more detailed analysis is required.

For example, our recent studies have shown that the exchange rate of W_s_ in the S_2_ state depends on the equilibria between the S_2_^A^, S_2_^AW^, S_2_^BW^ and S_2_^B^ states of the Mn_4_CaO_5_ cluster (Scheme 2).^44,46,55^ This allows O5 to reach a terminal position on a Mn^III^ ion (Mn4) and to be exchanged with bulk water. For W_f_ the situation is less clear as previous data allow for two options: either the W_f_ exchange rate also depends on conformational equilibria, or its exchange is limited by diffusion of bulk water through the channels leading to the Mn_4_CaO_5_ cluster. Knowing which exchange mechanism applies may help identifying W_f_ and thus for experimentally elucidating the mechanism of water oxidation.

If conformational changes determine the exchange kinetics, then the Mn4-ligated W2 must be W_f_ because these equilibria only affect the exchange of W2 and not that of the Ca-ligated W3. The absolute rate for W_f_ exchange, which is orders of magnitude slower than previously reported for water ligands of Ca ions and too fast for a water ligand of a Mn(iv) ion, can in this case be explained *via* the equilibrium between the S_2_^A^ and S_2_^B^ states, because in the S_2_^B^ state Mn4 has the oxidation state Mn(iii) that allows for rapid water exchange (Mn(iii) is exchange-labile; Mn(iv) is exchange inert – for discussion see ref. 9,18 and 57). Binding to Mn would also explain the insensitivity of the W_f_ exchange rate to Ca/Sr substitution.

If diffusion of water through channels determines the exchange kinetics, then the Ca-ligated W3 would remain an option for W_f_, because this limitation would explain that W_f_ exchange is comparatively slow for a Ca-bound water ligand and that its exchange is unaffected by Ca/Sr substitution. In this case, it would be impossible to distinguish W2 or W3 as the fast exchanging substrate in wild-type PSII in the S_2_ state on the basis of substrate water exchange rates, unless some treatment shifted the equilibrium between S_2_^A^ and S_2_^B^ strongly towards S_2_^A^, as this would keep W2 bound to an exchange-inert Mn(iv) ion, leading to a very slow exchange of W2.

In the following, we will first analyze if the faster water exchange in D61A-PSII is due to a shift of conformational equilibria, or if the truncation of this amino acid from aspartate to alanine increases water accessibility to the catalytic site. Subsequently, we will elucidate the consequences of this result for (i) understanding the exchange rates in the other S states and (ii) the assignment of W_f_. Finally, we will discuss the remaining options for the mechanism of water oxidation.

### W_f_ exchange in the S_2_ state

In the S_2_ state, W_f_ exchanges significantly faster than in WT-PSII in the majority of D61A-PSII centers (85%; Table 1). If a shift in conformational equilibria accounts for this observation, the Mn4-bound W2 would be the most likely assignment for W_f_, as outlined above. In this case, the D61A mutation would induce a change in the conformational equilibria of the Mn_4_CaO_5_ cluster towards the S_2_^B^ state (or another S_2_^HS^ state), because this allows W2 to exchange much more readily compared to WT-PSII.^46^ Therefore, a faster exchange of W2 in D61A-PSII would imply that the activation barrier for reaching the S_2_^B^ state would be lower and/or the relative stability of the S_2_^B^ state would be increased in the mutant. However, previous experimental data show that a stabilization of the HS S_2_^B^ state can be excluded, as only the LS S_2_ multiline signal was observed in the D61A-PSII samples and its signal intensity was comparable to that of WT-PSII (see ESE-EPR spectra in ref. 75). This is supported by theoretical calculations that find the equilibrium between the S_2_^A^ and S_2_^B^ state unchanged or even slightly shifted in favor of the S_2_^A^ state.^71^ These calculations also indicate that in D61A-PSII one proton is lost from the W1/W2/Mn4 site of the cluster.^71^ Such a proton loss would slow the W2 exchange. In conclusion, the direct chemical changes that can be expected to occur would either leave the water exchange the same or likely even slow the exchange of W2, the opposite to what is observed experimentally for W_f_ exchange. This analysis shows that a shift of the conformational equilibrium between S_2_^A^ and S_2_^B^ cannot explain the present data.

On this basis, we conclude that the exchange of W_f_ by isotopically labelled bulk water must be slowed by a steric constraint in all the channels that supply substrate to the Mn_4_CaO_5_ cluster in WT-PSII.^57^ The D61A mutation then appears to remove one of these diffusion barriers so that W_f_ exchange can occur at the experimentally observed faster rate. Indeed, barriers for water transport were described previously for all channels, and D1-D61 was identified as forming a barrier for water access together with D2-K317 and Cl1.^61^ We propose that shortening D1-D61 *via* the D61A mutation creates a void that is filled by one or two water molecules, which promotes faster water diffusion to the Mn_4_CaO_5_ cluster. This idea is in line with a recent theoretical study that shows water redistributions and faster movements of water molecules in the D61A mutant.^76^

### Model for W_f_ exchange *via* the Cl1 channel in the S_2_ state

Our data strongly indicate that D61 forms a steric barrier for water access to the catalytic site that contributes to limiting the rate of W_f_ exchange in the S_2_ state. However, comparison of the measured water exchange rates to water transport rates estimated from MD simulations appears to contradict this conclusion: in WT-PSII, the rate for W_f_ exchange is about 80 s^−1^ (at 283 K), while barriers of 10–14 kcal mol^−1^ calculated for all channels for moving a water molecule from the bulk to the Mn_4_CaO_5_ cluster would predict exchange rates up to a 1000-fold faster than our observation (see ESI TEXT 3†).^61^ However, the two processes are not directly comparable. MD simulations of water movements always employ a force to achieve concerted or directed water movement along a certain trajectory. This force can be provided for example by inserting extra water molecules near the Mn_4_CaO_5_ cluster, or by pulling water molecules through the channels at a constant velocity.^61^ By contrast, isotopic equilibration involves random swapping of neighboring water molecules driven by thermal energy. It thus requires many swapping events to reach isotopic equilibrium between an inner water pool and bulk water.

As D61 is located at a branching point of the O4 channel and the Cl1 channel, the faster water access may occur through either or both of these channels. The O4 pathway (channel 2 in ref. 61) has been proposed to facilitate substrate water entry^36,77–80^ because binding sites for the substrate analogues ammonia^75,81–83^ and methanol^78,84–86^ are located in the vicinity of Mn4, O4, and D1-D61. Also, the D1 residue at position 87, which is near the origin of the O4 pathway, is Ala in spinach and Asn in cyanobacteria, a fact that appears to correlate with the finding that methanol has a much larger effect on EPR signals of the Mn_4_CaO_5_ cluster of plants than cyanobacteria.^77,78^ However, other reports find that the O4 channel is rather narrow and possibly unsuitable for water transport and instead favor the Cl1 channel (or O1 channel) as main water access pathway.^60,63,84,87^

**Scheme 3:**
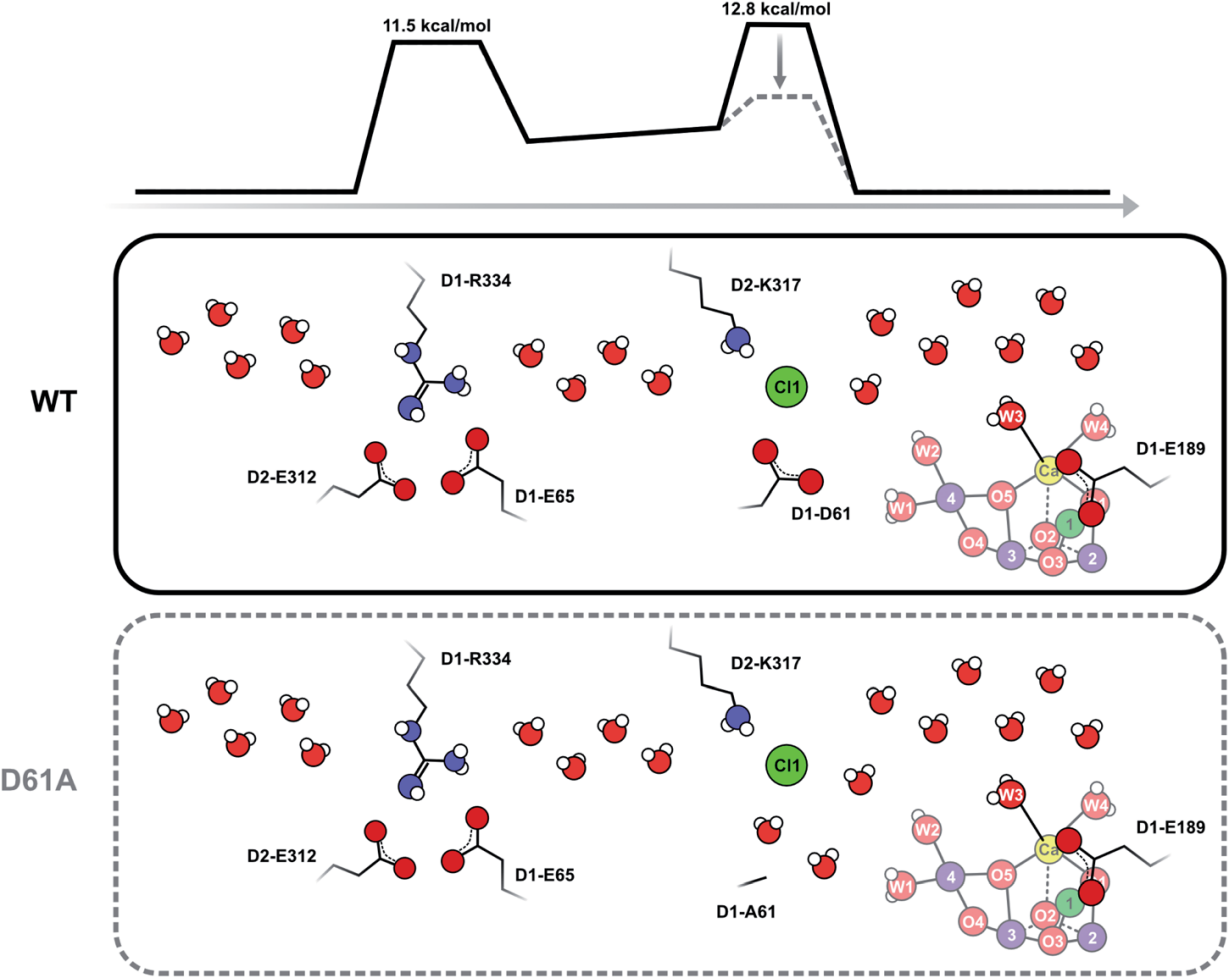
Schematic view of the Cl1 channel that is based on crystal structure information^15,17,21^ and MD simulations.^61^ The energy barriers have been slightly adjusted from the previous estimates in accordance with the present results. It is proposed that D1-D61 together with D2-K317 and Cl1 participate in forming the inner barrier that determines the rate of water exchange in WT-PSII. Shortening of D1-D61 to alanine reduces the second barrier by creating new water binding sites. In this case, the outer barrier, formed by the D1-E65, D2-E312 and D2-R334 residues, becomes rate limiting. As this outer barrier has a lower height, water exchange becomes faster in D61A-PSII. Barrier heights are estimated from the measured exchange rates as described in ESI Text 3.† These estimates assume that the frequency factor of the Eyring equation is 1.0; we note that it is possible that in reality a lower frequency factor should be used for the exchange processes, which could lower the barrier height.

To test the validity of our conclusion we examined the expected substrate water exchange rates through the shorter (25 Å) arm of the Cl1 channel (‘channel 1’ in ref. 61). This channel is reported to have two barriers: the first is formed by the D1-E65/D1-R334/D2-E312 triad and has a barrier of 11.5 kcal mol^−1^, while the second is formed by D1-D61, D2-K317 and Cl1 and has a barrier of 7 kcal mol^−1^ in the inward direction, and about 11 kcal mol^−1^ in the outward direction (Scheme 3). Using these parameters, we constructed a model that included two significant barriers, while other waters can exchange essentially freely. Eight water molecules, including W3 (but not W1, W2 and W4), formed the inner pool. To further simulate the water channel characteristics observed in crystal structures,^6,15,17,61^ four water molecules were placed between the two barriers, and five crystal waters are in rapid exchange with bulk water (Scheme 3; ESI Text 3†). We achieved excellent agreement with our experimental data by assuming that the inner barrier, formed by D1-D61, D2-E317 and Cl1, has an energy of 12.8 kcal mol^−1^, and the barrier closer to the bulk formed by D1-E65, D1-P66, D1-V67 and D2-E312 has a height of 11.5 kcal mol^−1^ (Fig. S5 and Table S3†). The inner barrier is slightly higher than determined for the outward direction by MD simulations, but this value is presumably within the accuracy of the MD method. It is also possible that the barrier for swapping two water molecules is actually higher (or the frequency factor lower; see SI Text S3) than for pulling water molecules through a channel,^61^ as this process requires two water molecules to pass each other in a bottleneck. This simulation thus shows that our proposal of an access limitation of the fast water exchange in the S_2_ state is realistic.

### W_f_ exchange in the S_0_, S_1_ and S_3_ states of the majority of D61A-PSII centers

In the S_3_ state, W_f_ exchange is slower than in the S_2_ state and thus no longer controlled by water access. This implies that W_f_ is now more tightly bound, in line with the suggested movement of W_f_ into the Ox or O5 positions (Scheme 1). Because in the S_3_ state all Mn ions are in oxidation state Mn(iv), the rate of the fast water exchange is limited instead by the redox equilibrium between the S_3_^AW^Y_Z_ and S_2_^AW^Y_Z_˙ states.^46,55^ The exchange of W_f_ in the S_3_ state most likely occurs by a reversal of the insertion pathway (Scheme 1).

The exchange of W_f_ becomes observable for the first time in the S_2_ state, which might be taken as indication of a faster exchange of W_f_ in the S_0_ and S_1_ states. This would be inconsistent with an S state independent water access barrier. However, simulations show that the required dark-times of 10 ms between the subsequent flashes employed for producing O_2_ (Fig. S2†) are long enough to scramble basically all isotopic information regarding the exchange kinetics of W_f_ in S_0_ and S_1_ (see^44^ and ESI Text 2†). Thus, the unresolved W_f_ exchange in the S_0_ and S_1_ states is consistent with an S state independent water access barrier; that is, with a diffusion limited exchange in the S_0_, S_1_ and S_2_ states, and thus with W3 or W2 as W_f_ in these states.

### W_s_ exchange in the S_2_ state of the majority of D61A-PSII centers

While improved substrate access provides a satisfying rationale for the unresolved and therefore more than 3-fold faster W_f_ exchange in D61A-PSII, it does not explain the 15-fold faster exchange of W_s_ in the dominant fraction of D61A-PSII centers. We recently observed a similar acceleration in WT-PSII at pH 8.6 and in Sr-PSII core complexes at pH 8.3.^46^ In this earlier study, the accelerated exchange correlated well with a stabilization of the S_2_^HS^ state, indicating that at normal pH the conversion from the S_2_^LS^ configuration into the S_2_^HS^ configuration is limiting the rate of W_s_ exchange. We assigned the alkaline-induced S_2_^HS^ state to the S_2_^AW^ state, as this state allows an easy transition into the S_2_^BW^ state (Scheme 2) in which O5 exchange can occur rapidly.^44,46,55^ As discussed above, the situation is different in the D61A mutant because the available data clearly exclude the stabilization of a S_2_^HS^ form.^71,75^ However, since water exchange in the S_2_^BW^ state is presumably very fast, and the S_2_^AW^ to S_2_^BW^ transition also has a comparatively low barrier,^42,55^ a similar acceleration of W_s_ exchange can be achieved by lowering the barrier for the rate limiting transformation of S_2_^A^ into the S_2_^AW^ state.

As shown in Scheme 1, water insertion during the S_2_ → S_3_ transition requires the deprotonation of W3. The same is true for the formation of S_2_^AW^ from S_2_^A^, which likely occurs in a similar fashion to mechanism A in Scheme 1. In the S_2_ state of WT-PSII, this proton needs to be transported away from the positively charged catalytic site into the bulk phase. In D61A-PSII, W1/W2 have collectively lost one proton,^71^ and should thus be able to transiently act as a nearby base that accepts the W3 proton during S_2_^AW^ and S_2_^BW^ formation. We propose that this lowers the energy barrier for S_2_^AW^ formation enough to allow the observed 15-fold increase in W_s_ exchange rate. That the breakage of an OH bond is rate determining for O5 exchange in the S_2_ state is supported by the H/D isotope effect of 1.9 ± 0.2 determined for W_s_ exchange in the mutant (Fig. S3; Table S2†).

### S_2_ state water exchange in the minority of D61A-PSII centers

We found that in about 15% of the D61A centers the exchange rates for W_f_ and W_s_ were similar to each other and to W_s_ exchange in WT-PSII (Table 1). This means that W_f_ exchange in this minority fraction was 10-fold slower than W_s_ exchange in the majority fraction, 60-fold slower than W_f_ exchange in WT-PSII, and more than 200-fold slower than W_f_ exchange in the majority fraction. By contrast, W_s_ exchange was slowed only 2–3 fold compared to WT-PSII, but nearly 40-fold relative to the majority fraction.

We see two options to explain the slow and comparatively similar rates of exchange of W_f_ and W_s_ in this fraction of the D61A-PSII. Firstly (Option 1), in these centers the D61A mutation induces a secondary structural change that restricts the water access at a different point of the channel even more than in WT-PSII. For example, if the Cl1 channel would be the dominant substrate entry pathway, such a secondary structural change might occur at the D1-E65/D1-R334/D2-E312 triad, which was suggested previously to be another bottleneck for water transport through the Cl1 channel.^61^ As this triad provides a rather narrow path for water, a small change in protein conformation or dynamics may be enough to further restrict water passage. As the D1-D61A mutation is only 4 amino acids away from D1-E65, such an allosteric effect cannot be excluded. Secondly (Option 2), both W3 and W2 serve as W_f_, but in different populations of D61A PSII centers, with one serving as W_f_ in the majority fraction and the other serving as W_f_ in the minority fraction. This idea is motivated by the similar rates of exchange found for W_f_ and W_s_ in the minority fraction of D61A-PSII, which suggest that their exchange may be limited by the same critical steps. This would be the case for W2 and O5, as substrate exchange *via* the S_2_^A^, S_2_^AW^ and S_2_^BW^ route places both at terminal positions of Mn4(III) in the S_2_^BW^ state. This option would indicate a substantially increased barrier for the S_2_^A^ to S_2_^B^ conversion in the D61A-PSII (from 6–10 kcal mol^−1^ in WT^41^ to >16 kcal mol^−1^).

### W_s_ exchange in the S_3_ state

In contrast to the S_2_ state, no heterogeneity is observed in the W_s_ exchange in the S_3_ state. Thus, if it is correct that W_f_ is different for the two fractions in the S_2_ state (Option 2, above), then rearrangements of the cluster must happen during the transition from S_2_ → S_3_ that bring the substrates into the same binding sites in the S_3_ state. This would indeed be possible, if in one fraction of PSII centers W3 and O5 are the substrates in the S_2_ state and W3 is inserted into the Ox position in the S_2_ → S_3_ transition (Scheme 1A), while in the other fraction W2 and O5 are the substrates that reach the same two binding sites *via* a pivot insertion (Scheme 1C).

Exchange of W_s_ (O5) in the S_3_ state thus occurs most likely *via* the S_2_^AW^Y_Z_˙ state, which further transforms into the S_2_^BW^Y_Z_˙ state where O5 is bound in a terminal position at Mn4 and can be replaced *via* the S_2_^B^Y_Z_˙ intermediate (Scheme 2).^44,46,55^ The much slower exchange of W_s_ in the S_3_ state of D61A-PSII as compared to WT-PSII indicates that in the D61A mutant the back donation of an electron from Y_Z_ to the Mn_4_CaO_6_ cluster is less efficient than in WT-PSII.^55^ One alternative for O5 exchange in the S_3_ state would be its exchange *via* the S_3_^BW^ and S_3_^B^ states (Scheme 1).^44,46^ In this case, the slowed W_s_ exchange implies a destabilization of one or both of these states as compared to the S_3_^AW^ state.

### Absence of effects of E189Q mutation

The analyses of recent XFEL studies favor water delivery *via* the O1 channel, and some of the authors suggest a gating of water access by the observed movement of E189 during the S_2_ → S_3_ transition.^14,15,17,21^ As the W_f_ exchange rates in the S_2_ and S_3_ states are nearly identical between WT-PSII and E189Q-PSII, the present data do not support a role of E189 as gate keeper for water access to the Mn_4_CaO_5/6_ cluster, at least not during water exchange in the studied semi-stable S states. This is in line with the MD calculations by Vassiliev, which suggest that D1-E329, D1-D342, CP43-V410 and CP43-T412 form the main bottleneck for water transport through the O1 channel system,^61^ and thus any subtle effects of E189 would be masked.

### Relation of water exchange and water binding

While our data show that in D61A-PSII water exchange occurs through the O4 and/or one or both branches of the Cl1 channel, they do not reveal which of the channels, including the O1 channel, has the lowest barrier in WT-PSII. Furthermore, it is important to note that water binding during the S_2_ → S_3_ transition is a fundamentally different and much faster (100–400 μs)^15,17^ process than water exchange in the S_2_ and S_3_ states (10–500 ms). During water binding, a nearby water attaches to an open binding site of the cluster and thereby initiates a bucket brigade of refilling vacant sites, while reaching the isotopic equilibrium with bulk water requires full equilibration of all exchangeable water molecules in the channels and around the catalytic site. Thus, our present data do not identify through which of the three water channels the substrate water is delivered in WT-PSII.

### Is the control of water access functionally important?

It has previously been hypothesized that regulation of substrate accessibility is crucial to minimize side reactions that would lead to the production of reactive oxygen species at the Mn_4_CaO_5_ cluster.^88,89^ This hypothesis assumed that in intact PSII complexes only substrate water can interact in a specific way with the Mn_4_CaO_5_ cluster. Recent crystal structures have shown that the Mn_4_CaO_5_ cluster is surrounded by several additional water molecules. Nevertheless, the present data and the previous calculations by Vassiliev^61^ show that water access is not completely free. This somewhat regulated access likely evolved to stabilize the Mn_4_CaO_5_ cluster, and to allow for the formation of a highly specific hydrogen bonding network, which is crucial for removing protons from substrate water during the water oxidation reactions. By contrast, the access of water is fast when compared to the maximal turnover frequency of PSII, which is limited by the acceptor side reactions of PSII to about 50 O_2_ s^−1^ (20 ms),^90^ while water is delivered through the channels with a time constant in the order of 100 μs.^61^ Interestingly, the time constant for water delivery is in the same order as that for water binding during the S_2_ → S_3_ transition. It might thus be speculated that the restriction of water access is a compromise between excluding other redox active molecules and ions from the Mn_4_CaO_5_ cluster, while allowing fast enough water access to promote efficient S state turnover. This idea is supported by the finding that partial dehydration of PSII increases the misses specifically of the S state transitions that involve binding of water molecules.^91^ Similarly, addition of the water analog methanol increases the miss parameter and allows the observation of a water deprived S_3_ state.^92,93^

### Implications for the mechanism of water oxidation

The significance of the present results is that they remove the strongest arguments against the assignment of W_f_ to W3 in the S_2_ state, namely (i) the indifference of the W_f_ exchange rate to Ca/Sr substitution and (ii) the significant mismatch with reported exchange rates for water ligated to Ca.^46,94^

**Scheme 4:**
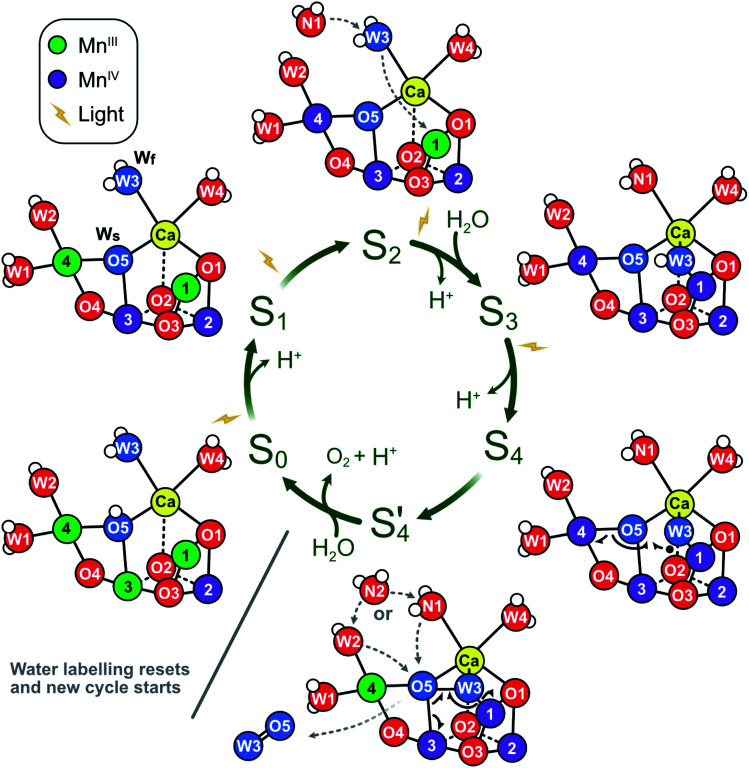
Proposed molecular Kok cycle illustrating the binding of the two substrate waters W_f_ and W_s_ in the various S states. The center shows the traditional S state scheme indicating water binding as well as proton and dioxygen release, while the outer circle depicts schematically the corresponding dominant structures of the Mn_4_CaO_5/6_ complex based on X-ray crystallography^6,15,17,21^ as well as calculated structural models of key intermediates during O–O bond formation.^12^ In dark-adapted PSII, the reaction cycle starts with the S_1_ state that has two Mn^III^ and two Mn^IV^ ions and in which all bridges are deprotonated.^10^ During the S_1_ → S_2_ transition, Mn4 is oxidized. While the S_2_^A^ state is in equilibrium with other conformations (see Scheme 2), it is proposed that W3 is inserted directly into the Ox binding site between Ca and Mn1, concomitant with Mn1 oxidation and the binding of a new water, W_N1_, to the W3 site (dashed grey arrows; for details, see Scheme 1A).^17^ In S_3_, the dominant state is S_3_^AW^. Upon further oxidation, the S_3_^AW^Y_Z_˙ state is formed, which under proton release converts into the S_3_^AW^Y_Z_˙’ state (lag phase; not shown).^103^ This may be coupled to unknown rearrangements within the H-bonding network of the OEC. Only thereafter, the Mn_4_CaO_6_ cluster can be oxidized to S_4_. Instead of Mn oxidation, S_4_ state formation involves the oxidation of the fast substrate water, indicated by a black dot on W3 (in the Ox position).^12^ By rearranging the electrons of the chemical bonds (black half-arrows), the S_4_ state rapidly converts into the S_4_’ state, which contains a complexed peroxide. The further conversion of S_4_’ into S_0_ + O_2_ requires the binding of one water and the release of a proton. We suggest that a pre-bound water ligand (W2 or W3) fills the empty O5 binding site,^9^ and that this ligand is concomitantly replaced by a new water (W_N2_; dashed grey arrows). In the S_0_ state, the O5 bridge is protonated, in line with the faster exchange of W_s_ and spectroscopic data.^4,104,105^ Oxygen atoms are labeled red, and the two substrate ‘waters’ are shown in blue. Hydrogen atoms are shown as small white spheres (protonation states based on S_2_ state assignment in ref. 106).

The present data are fully consistent with O5 as slowly exchanging substrate water W_s_, and W2 or W3 as fast exchanging substrate water W_f_. A further distinction between W2 and W3 as W_f_ is not possible on the basis of substrate water exchange data alone because the rate limitation provided by the barriers in the channels obscures small perturbations such as Ca/Sr substitution that could otherwise be used to distinguish the binding sites. However, other recent experimental data favor W3 over W2 as substrate water. FTIR experiments by the groups of Noguchi and Debus have provided evidence for the involvement of W3 in water binding during the S_2_ → S_3_ transition.^11,16,95^ Similarly, femtosecond X-ray crystallography measurements have revealed that the largest changes in water positions during this transition occur in the O1 channel that leads to the Ca site and found no evidence for the predicted closed cube S_2_^B^-like intermediate that would be required if W2 were the fast substrate (Scheme 1).^15,17^ By contrast, the support for W2 is mostly based on substrate analogs like methanol or ammonia,^75,77–83^ which we regard as more indirect. On this basis, we propose that O5 and W3 are the two substrate water molecules under normal circumstances, but that W2 may serve as the fast exchanging substrate under some circumstances, such as in a minority of D61A PSII centers. The resulting experimentally supported ‘molecular S state cycle’ is summarized in Scheme 4.

Presently no experimental data are available that allow to determine the actual O–O bond formation mechanism during the S_3_ → S_4_ → S_0_ transition, but the present data are fully consistent with the best worked out theoretical mechanism for O–O bond formation, which involves oxo-oxyl radical coupling between oxygens in the O5 and Ox binding sites *via* a low-energy path paved by favorable spin paring.^12,53^

However, the recently revived idea that the formation of a peroxidic intermediate (<5–10%) in the S_3_ state is required for further oxidation to the S_4_ state cannot be excluded on the basis of our present data (Fig. S1E and F†),^1,21,27,28^ because the same substrates and main state conformations are involved, and such a small equilibrium population of a peroxidic intermediate would easily escape detection by, for example, femtosecond X-ray crystallography. Nevertheless, a very recent theoretical study considers a peroxidic intermediate in the S_3_ state as unlikely.^96^ By contrast, our substrate water exchange data are inconsistent with nucleophilic attack mechanisms between W3 and W2,^51,97–99^ and geminal coupling between W2 and O5 at Mn4 (ref. 30, 100) (for details see ESI Text 4 and Fig. S1†).

## Conclusions

In this study, we demonstrate that the fast water exchange in the S_0_, S_1_ and S_2_ states is rate limited by specific diffusion barriers in all the channels connecting bulk water with the Mn_4_CaO_5_ cluster in PSII, and that the D61A mutation reduces one of these barriers so that W_f_ exchange is accelerated. This finding removes previous arguments that appeared to exclude W3 as the fast exchanging substrate water. Combining our present results with recent FTIR and XFEL data supporting the insertion of W3 into the Ox position during the S_2_ → S_3_ transition,^11,15–17,95,101^ now make W3 the prime candidate for W_f_. As our previous experiments identified O5 as the slow substrate water,^9,18,44,46,54^ this study clarifies the fate of the substrate waters during the S state cycle, and thereby limits the possible mechanisms for O–O bond formation to a few that all involve coupling between O5 and W3, while they are bound in the O5 and Ox positions of the S_3_^AW^ or S_4_^AW^ states (Scheme 4).

## Data availability

All relevant data is presented in the paper and ESI.† Raw data is available upon request by email to JM.

## Author contributions

CDL, RJD and JM conceived and designed the research; CDL, CJK and PC performed the research; CDL and JM analyzed the data; CDL, RJD and JM wrote the paper with input from all authors.

## Conflicts of interest

There are no conflicts to declare.

## Supplementary Material

SC-012-D1SC02265B-s001
