# Supplementary material for: The exchange of the fast substrate water in the S2 state of photosystem II is limited by diffusion of bulk water through channels – implications for the water oxidation mechanism
Source: Chem Sci. 2021 Sep 1;12(38):12763–75. doi: 10.1039/d1sc02265b (PMC8494045; doi:10.1039/d1sc02265b)
Supplement: SC-012-D1SC02265B-s001 [file SC-012-D1SC02265B-s001.pdf]

Electronic Supplementary Material (ESI) for Chemical Science.
This journal is © The Royal Society of Chemistry 2021

The exchange of the fast substrate water in the S_2 state of photosystem II is limited by diffusion of bulk water through channels – implications for the water oxidation mechanism

Casper de Lichtenberg,[a,b] *Christopher J. Kim,*[c] *Petko Chernev,*[b] *Richard J. Debus,**[c] *& Johannes Messinger**[ab]

[a]Department of Chemistry, Umeå University, Linnaeus väg 6 (KBC huset), SE-901 87, Umeå, Sweden
[b]Molecular Biomimetics, Department of Chemistry – Ångström Laboratory, Uppsala University, POB 523, SE-75120 Uppsala, Sweden
[c]Department of Biochemistry, University of California, Riverside, California 92521, United States

Supplementary Information

SI Text 1: *Analysis of the substrate water exchange rates*

The exchange rates (k_{f1}, k_{f2}, k_{s1} and k_{s2}) for the fast and slow substrate waters were determined by a simultaneous fit of the m/z 34 and the m/z 36 data to **Equations 1 and 2**.[1-3] The parameter *a* is the ratio of the amplitudes of the fast and slow phases of substrate water exchange in the $^{34}O_2$ data. The parameter *a* was determined from the initial enrichment (α_{in}) and the final enrichment (α_f) as shown in **Eq. 3**. [1] The initial enrichment was found to be 0.7%, i.e. slightly elevated over natural abundance as a result of leakage from the syringe tip.

The parameter *b* was determined through a preliminary fit of the m/z 36 data and essentially represents a partition of different populations of the PSII core particles in which W_s and W_f exchange with either rate 1 or rate 2. The parameter *b* was only necessary for fitting the D61A-PSII data, and was set to 0 for both WT- and E189Q-PSII.

We note that in the data of W_f exchange in the S_2 state there is a contribution from the exchange of W_f that occurs in the S_3 state during the dark time of 10 ms between the 2nd and 3rd flash that are employed to produce oxygen (**Figure S2**). This generates a mixing-time dependent offset *c* in the exchange phase of W_f, which can be calculated from k_f in the S_3-state and the dark time (t_{dark}) between the 2nd and 3rd flash (**Eq. 4**). To account for the exchange of W_f in the S_3 state, the time-dependent offset was included in the fit of the m/z 34 data of the S_2-states as shown in **Eq. 1**. Values of the parameters *a*, *b* and *c* are given in **Table S1**.

$$m/z\ 34 = a \cdot \left(b * \left(1 - c * e^{-k_{f1} \cdot t}\right) + (1-b) * \left(1 - c * e^{-k_{f2} \cdot t}\right)\right) + \\ (1-a) \cdot \left(b * (1 - e^{-k_{s1} \cdot t}) + (1-b) * (1 - e^{-k_{s2} \cdot t})\right) \quad (1)$$

$$m/z\ 36 = b * (1 - e^{-k_{s1} \cdot t}) + (1-b) * (1 - e^{-k_{s2} \cdot t}) \quad (2)$$

$$a = \frac{\alpha_f \cdot (1-\alpha_{in}) + (1-\alpha_f) \cdot \alpha_{in}}{(1-\alpha_f) \cdot \alpha_f \cdot 2} \quad (3)$$

$$c = 1 - \left(1 - e^{-k_f^{S3} \cdot t_{dark}}\right) \quad (4)$$

Corrections for sample dilution and isotopic enrichment were not required,[2] since all measurements were performed after full mixing was achieved (> 6 ms). This mixing time will lead to only a small underestimation of the actual W_f exchange rate, which is 10-fold slower.

SI Text 2: *Resolution of W_f exchange in the S_1 states*

To test if it is feasible that W_f exchanges with the same rate in the S_1 state as it does in the S_2 state due to the same access barriers in the channels leading to the Mn_4CaO_5 cluster, we estimate here the amplitude of the fast phase that would be resolvable, i.e. remains non-exchanged. The exchange rate of W_f in the S_1 state is masked, at least in part, by further exchange of W_f in the dark times between the 3 flashes to produce O_2 after the end of the mixing time in S_1. These times were selected to be 10 ms in S_2 and 20 ms in S_3 in order to allow a reasonably large fraction of PSII to turn over and produce O_2, i.e. to have a good enough signal to measure. We also select a very short time (10 ms) of S_1 state exchange, in order to have a maximum amount on non-exchanged centers left, but to allow all water to mix (mixing time 6 ms). As exchange rates we employ for our estimate: $k_f(S_1) = k_f(S_2) = 84\ s^{-1}$ and $k_f(S_3) = 23\ s^{-1}$ (Table 1 main manuscript, and assuming same diffusion limitation in S_1 and S_2).

The population of PSII that will not equilibrate the W_f binding site ($^{ne}[W_f]$) with bulk water during the 10 ms in the S_1 state is then:

$$^{ne}[W_f]^{S1} = {}^{ne}[W_f]^{t=0} * e^{-kf(S1)*t} = 1 * e^{-84*0.01} = 0.43 \qquad (5)$$

Since the exchange rate and time are the same in the S_2 state, the non-exchanged W_f sites remaining after the 10 ms dark time between the first and second flash is:

$$^{ne}[W_f]^{S2} = {}^{ne}[W_f]^{S1} * e^{-84*0.01} = 0.43 * 0.43 = 0.18 \qquad (6)$$

We assume now 20 ms dark time in the S_3 state to allow quinone exchange on the acceptor side, which is resonable due tot he slower exchange in this state:

$$^{ne}[W_f]^{S3} = {}^{ne}[W_f]^{S2} * e^{-23*0.02} = 0.18 * 0.63 = 0.11 \qquad (7)$$

This shows that even with very short times between the flashes only about 10% of the fast phase (or about 6% of the total exchange; the amplitude of the fast phase 57% under our $H_2^{18}O$ enrichment conditions) would not be exchanged and thus could be observed. This explains why W_f exchange in S_1 has not yet been resolved.[4-6] For future experiments that aim to resolve k_f for the S_1 state, it would be imperative that the exchange rates of W_f exchange can be slowed in the S_2 and S_3 states, and that the dark times can be minimized, e.g. by pre-oxidation of the non-heme iron.

Note that the rates of W_f exchange reported in this study for S_2 are corrected for the additional exchange in the S_3 state (see above).

SI Text 3: *Estimation of time to equilibrate bulk channel water with bulk water*

A model of water traveling through a channel was constructed that consists of a sequence of (maximum) 17 positions that can be occupied either by a bulk/exchanged water molecule, or an original/non-exchanged water molecule (see **Scheme 3, main text**). The first position corresponds to the bulk and is always kept in the exchanged state; the remaining positions start the simulation in the non-exchanged state, with the last position corresponding to water bound to the OEC. A simulation time step is chosen

and fixed to 0.01 ms. At each time step, each pair of neighboring molecules in the chain has a chance to exchange positions. The probability of exchange is determined by a given free energy of activation (different for each pair of neighboring molecules). From the free energy, a rate constant of the exchange is calculated using the Eyring equation at 10°C with a transmission coefficient (κ) of one:

$$k = \frac{\kappa k_B T}{h}\, e^{-\frac{\Delta G}{RT}} \tag{10}$$

where ΔG is the energy of the barrier, k_B is the Boltzmann constant, T is temperature, h is Plank's constant, and R is the gas constant. The rate constant (in units exchanges per second) is then multiplied by the time step, giving the (average) number of molecules that are exchanged in each time step, which at values sufficiently smaller than one is assumed to correspond to the probability of an exchange happening ("probability value"), where a probability of one or higher always leads to exchange. At each time step, for each pair of neighboring positions, a random number between zero and one is generated and compared to the "probability value"; if the random number is smaller, then the two water molecules are exchanged. If the "probability value" is equal to or larger than one, then the exchange always happens, and thus different energy barriers that give such values give identical results. For example, at a time step of 0.01 ms, decreasing the energy of the barrier below 10.07 kcal/mol, which gives a "probability value" equal to one, has no effect on the simulation: the waters are always exchanged on each step. In this setup, more than one molecule can travel in the channel, a molecule can move more than once per time step, and a molecule can move forwards or backwards in the channel. The simulation (100 ms, 0.01 ms time steps) is run several hundred times and the occupancy of the last water position (using a value of zero for a non-exchanged and one for an exchanged molecule) as a function of simulation time is summed up to give an exchange time trace. A mono-exponential rise function with a fixed amplitude and time shift is fitted to the simulation to compare exchange rates to the experiment. All simulations were done in Excel.

A typical result of a simulation of the isotopic equilibration as suggested to occur in WT-PSII (blue) and kinetic fit thereof (black) is shown in **Figure S5**, and details of the simulation parameters are given in simulation 1 of **Table S3**. **Table S3** provides also additional simulations demonstrating for example the effect of the inner water pool size, or of changing barrier height.

SI Text 4: *Discussion of alternative mechanisms for O-O bond formation (see also ref [3])*

O-O bond formation in S_4^{BW} state: We cannot exclude that the S_3^{AW} state with O5 in the O5 binding site, $[O5]_{O5}$, and W3 in the Ox binding site, $[W3]_{Ox}$, converts in the lag phase after $S_3Y_Z^{\bullet}$ formation into the S_3^{BW} state, so that the oxyl radical is formed at the Mn4 site. This would allow O-O bond formation between $[O5]_{W2}$ and $[W3]_{O5}$ via **Mechanism B** in **Figure S1**. Such an option for oxyl radical coupling was proposed first on the basis of water exchange experiments,[7, 8] and more recently it was found to have a similar barrier as O-O bond formation by **Mechanism A** (**Scheme 4** and **Figure S1**).[9]

Nucleophilic attack: Nucleophilic attack between $[W_s]_{W2}$ and $[W_f]_{W3}$ was proposed before water insertion during the $S_2 \rightarrow S_3$ transition was known (**Figure S1 C**). The diffusion limitation of the exchange of the inner water pool proposed in the main text allows conceiving a possible realization *via* $[W_2]_{W2}$ and $[W_{N1}]_{W3}$. In this case, $[O5]_{O5}$ and $[W3]_{Ox}$ would need to refill the W2 and O5 positions during O_2 release. To explain the slower W_f exchange in the S_3 state, one would need to postulate that the D61 exchange barrier is higher in the S_3 state than in the S_2 state. Due to this in our view unrealistic assumption and the very good experimental and theoretical support for O5 as slow substrate water [7, 10-14] we exclude the nucleophilic attack mechanism. DFT calculations by Per Siegbahn have also discounted this mechanism as option for O-O bond formation in PSII.[15]

Geminal coupling: Geminal coupling at Mn4 (**Figure S1 D**) was recently proposed as an alternative to radical coupling.[16] This may be realized either with W3 insertion via **Routes B** or **A** in **Scheme 1**. In the former case, W2 and W3 would form the O-O bond, while in the latter it would be W2 and O5. O_2 formation between W2 and W3 is highly unlikely due to the good experimental and theoretical support for O5 as slow substrate water.[7, 10-14] With W2 and O5 as substrates in all S states, substrate exchange in the S_3 state would again require the equilibrium with Y_Z. The formation of the $S_2^{BW}Y_Z^{\bullet}$ state would allow both W2 and O5 to exchange. However, as both would be terminal ligands on Mn4(III), one would expect them to exchange with similar rates, which is in disagreement with the experimental observation. We note that a recent DFT study also excluded the geminal coupling mechanism on Mn4.[17]

O-O bond formation between W2 and O5: Water oxidation mechanisms involving the pivot water insertion (**Scheme 1C**) can lead to O-O bond formation between W2 and O5 via forming an S_3 state with W2 binding in the original O5 binding site, $[W2]_{O5,}$ and O5 binding in the Ox binding site of the S_3 state, $[O5]_{Ox}$. Structurally the S_3 state is the same, as would be reached by W3 insertion into the Ox site (**Scheme 1A**) so the same O-O bond formation mechanisms as discussed for $[O5]_{O5}$ and $[W3]_{Ox}$ are possible. In the S_3 state, the exchange of W_f would most logically occur in the $S_2Y_Z^{\bullet}$ state by reverting its insertion pathway. One problem with the pivot insertion of W2 into the O5 position during the $S_2 \rightarrow S_3$ transition is that reverting the step in the $S_2Y_Z^{ox}$ state brings W2 into a terminal position, but it remains unclear by which mechanism $[O5]_{Ox}$ shall exchange. The best option may be that $[O5]_{Ox}$ becomes W_f in the S_3 state in which it would exchanges via Ca (reverse of insertion A in **Scheme 1**), while $[W2]_{O5}$ would become W_s in the S_3 state. However, this poses the question why then water insertion does not happen by direct insertion of W3 to Ca (**Route A** in **Scheme 1**). We thus disfavor this option.

Inverted arrangement of W3 and O5: The insertion of W3 into the O5 position via Mn4 (**Figure 3B**) leads to the same 'inversion' of the binding sites during the $S_2 \rightarrow S_3$ transition as discussed above for the pivot mechanism. This also allows for the same O-O bond formation steps as discussed in the main text and shown in **Scheme 4** and **Figure S1 A, B, E, F**. Again, it is difficult to understand the exchange path for $[O5]_{Ox}$. In addition, as noted in the main text, serial crystallography experiments at various time points during the $S_2 \rightarrow S_3$ transition did not provide evidence for intermittent S_2^B formation,[18, 19] making insertion mechanisms **B** and **C** (**Scheme 1**) less likely.

Peroxidic intermediate: As discussed in the main Text, this option (**Mechanisms E** and **F** in **Figure S1**) cannot be excluded on the basis of present experimental data, because the presently accessible intermediates are identical or indistinguishable with regard to structure and Mn oxidation state due to the proposed low concentration of the peroxidic state in the S_3 state.

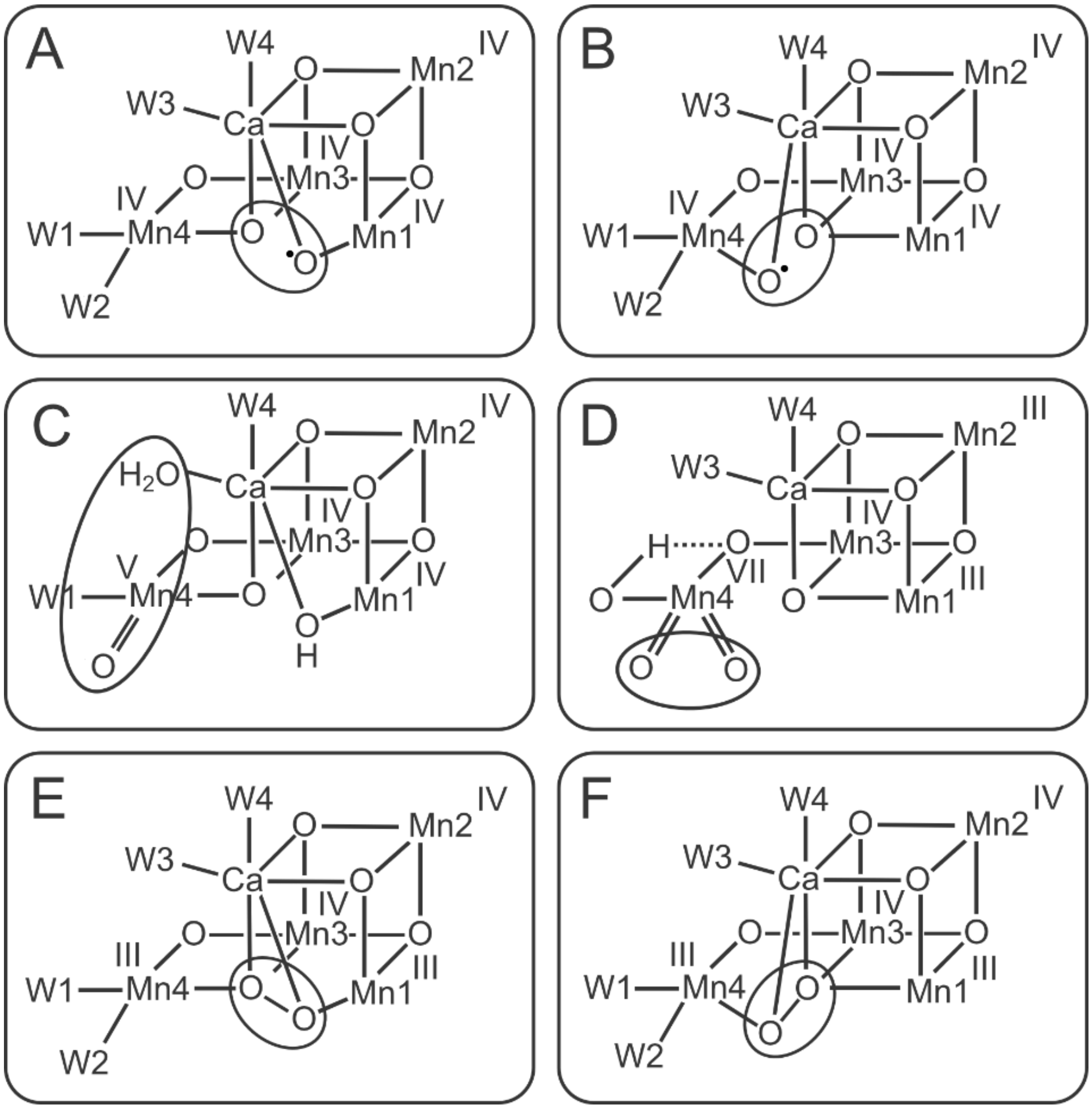

Figure S1: Proposed mechanisms for O-O bond formation in the S_4 state of PSII. A: radical coupling at the Mn1 site [12, 20], B: radical coupling at the Mn4 site [7-9], C: nucleophilic attack (adapted to the latest structural information) [1, 21-23]; D: geminal coupling [16, 24]; E, F: two options for a peroxidic state in the S_3 state [25, 26]. For these two latter mechanisms it is assumed that only this small fraction of peroxidic state can be oxidized to the S_4 state. For reviews on water oxidation mechanisms, see for example [27-29].

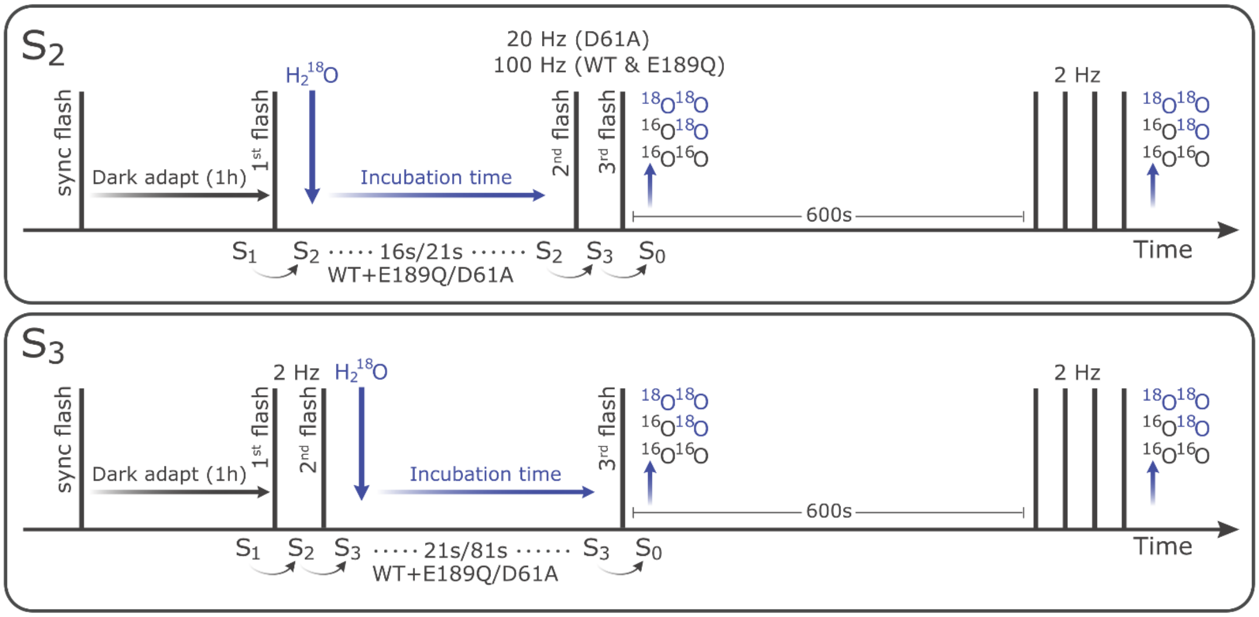

Figure S2: Flash-injection protocol employed for substrate water exchange measurements in the S_2 (top) and S_3 (bottom) states of photosystem II. The synchronization flash was applied to the dark-adapted samples in a glass vial to enrich the $S_1Y_D^{ox}$ state. The final flash group of four flashes was employed for monitoring the final enrichment of each experiment and to account for small changes in sample concentration or membrane permeability.

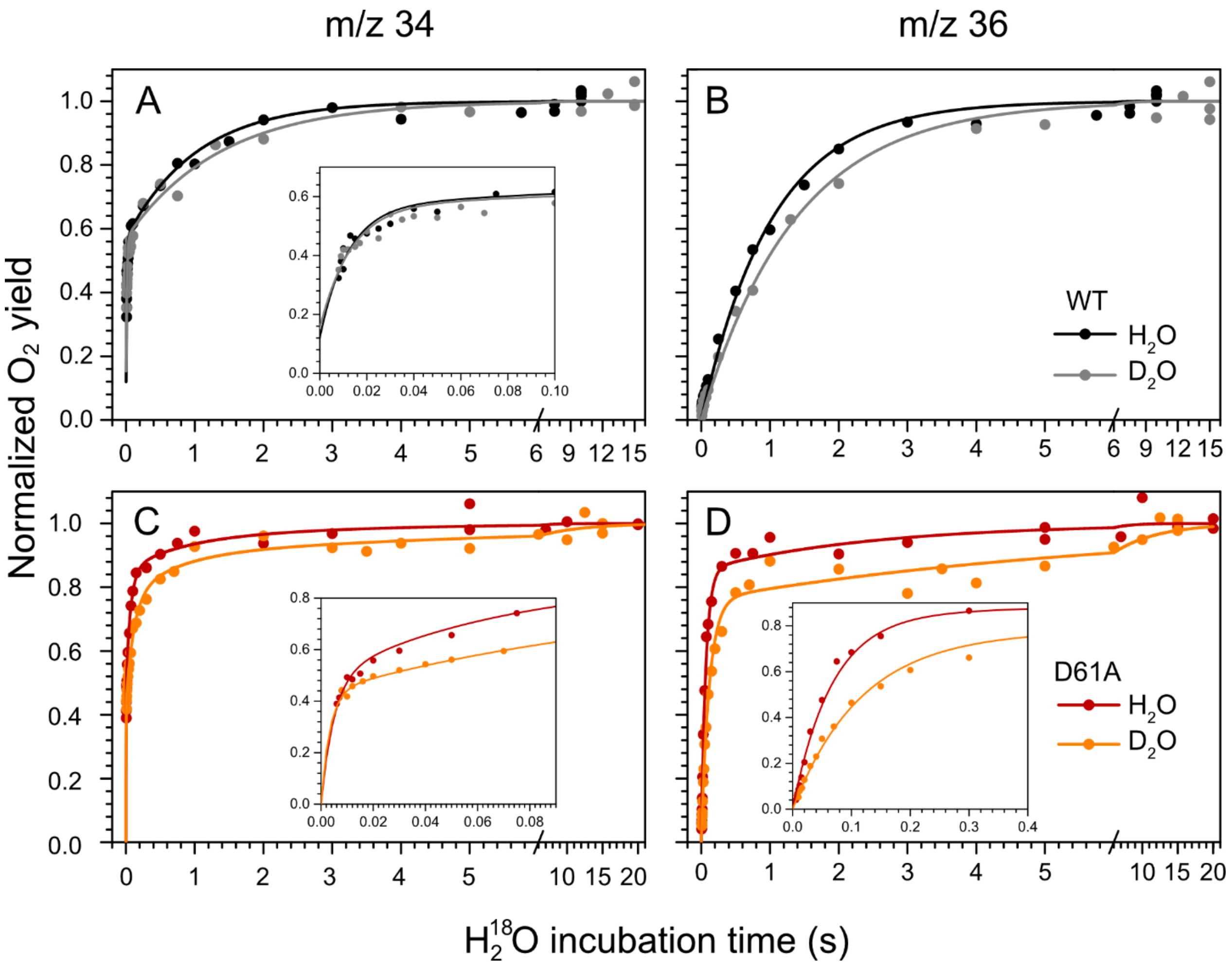

Figure S3: H/D-dependence of the substrate water exchange measurements in the S_2-state state of WT- (black/grey) and D61A- (red/orange) photosystem II core complexes of *Synechocystis* sp. PCC6803. The normalized oxygen flash yields of a double flash given after different incubation times with $H_2^{18}O$ in the S_2 state are plotted. A and C show the results for single labelled oxygen (m/z 34), while panels B and D those of double labelled oxygen (m/z 36). Dots represents measurements, while solid lines the results of kinetic fits (**Tables S1 & S2**). The inserts show an enlarged view of the fast exchange phase in the m/z 34 and m/z 36 data. Observe differences in the time scales. The data were recorded at 10ºC in buffer with either 100% H_2O or 88% D_2O (final concentration after $H_2^{18}O$ injection). The pL was 6.5.

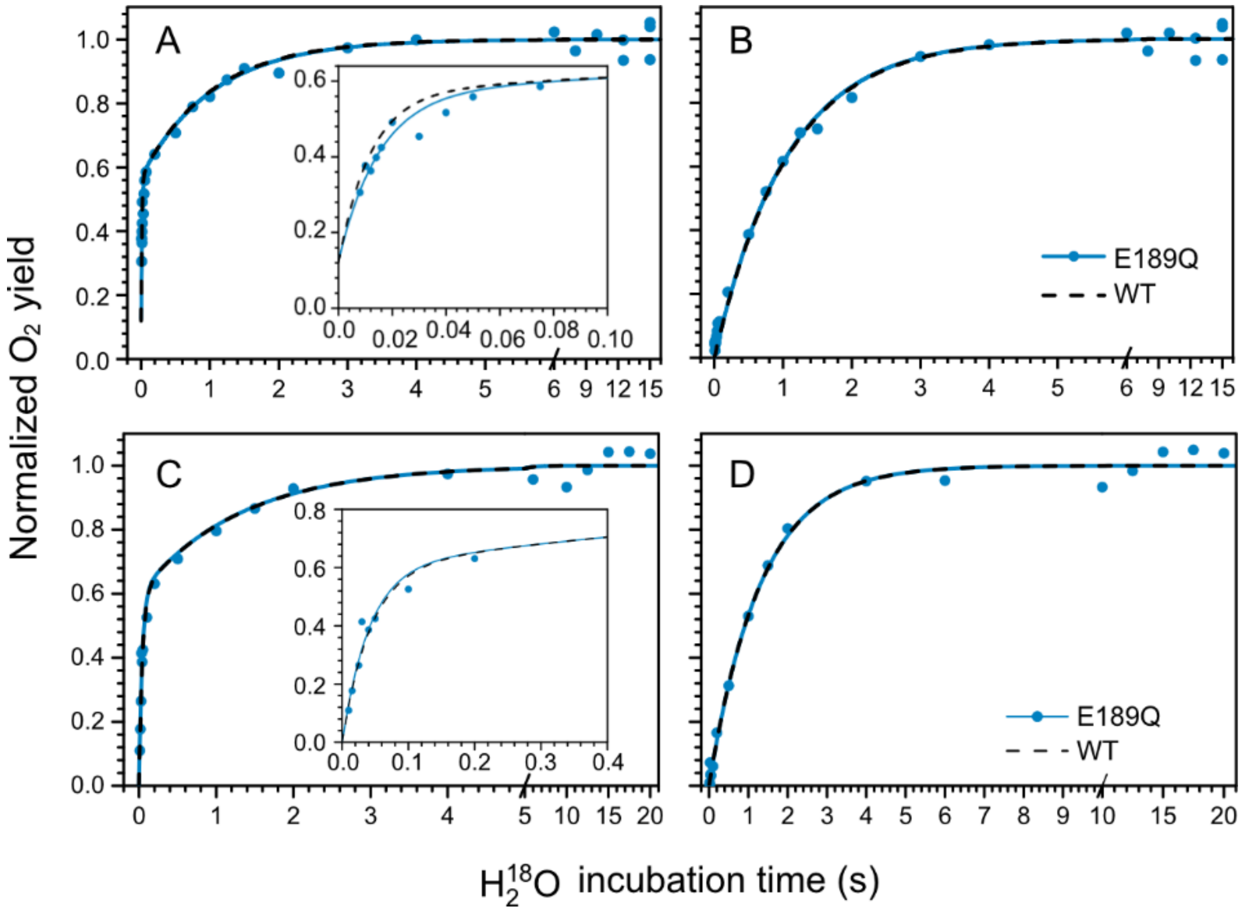

Figure S4: Substrate water exchange measurements in the S_2-state state (A, B) and S_3 state (C, D) of E189Q-PSII core complexes of *Synechocystis* sp. PCC6803. The normalized oxygen flash yields of a double flash (S_2) or single flash (S_3) given after different incubation times with $H_2{}^{18}O$ in the S_2 state are plotted. A and C show the results for single labelled oxygen (m/z 34), while panels B and D those for double labelled oxygen (m/z 36). Dots represent individual measurements, while solid lines the results of kinetic fits (**Table 1**). The fits of the WT-PSII substrate exchange (data in **Figures 2** and **3**; **Table 1**) are shown as a dashed line for visual comparison. The inserts show an enlarged view of the fast exchange phase in the m/z 34 data. Observe differences in the time scales. The data were recorded at 10°C, pH 6.5.

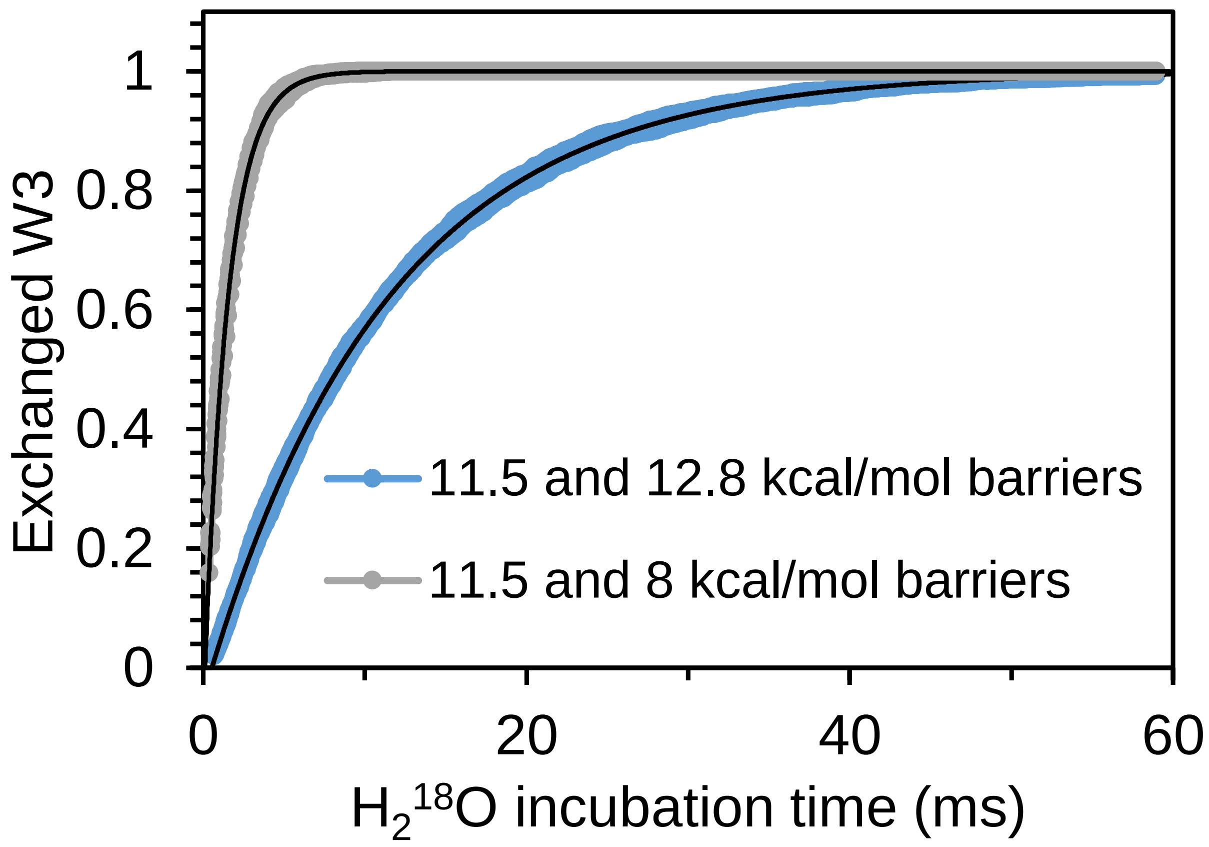

Figure S5: Typical results of simulation of the isotopic equilibration in WT-PSII (blue) and D61A-PSII (gray), with kinetic fits thereof (black). The WT-PSII simulation is based on two barriers, 11.5 kcal mol^{-1} and 12.8 kcal mol^{-1}, and 8 water molecules after the last barrier. The D61A-PSII simulation is based on 11.5 kcal mol^{-1} and 8 kcal mol^{-1} barriers (for further details see **Simulations 1** and **2** in **Table S3,** and **SI Text 3**).

SI Tables

Table S1: Parameters used in **Equations 1** and **2** for kinetic fitting of the $H_2{}^{18}O$ substrate exchange data.

	WT-PSII			D61A-PSII			E189Q-PSII		
**S-state \ parameter**	**a**	**b**	**c**	**a**	**b**	**c**	**a**	**b**	**c**
**S_2-H_2O**	0.57	0	0.79	0.57	0.85	0.98	0.57	0	0.78
**S_2-D_2O**	0.57	0	0.73	0.57	0.76	0.98	nd	nd	nd
**S_3-H_2O**	0.6	0	-	0.6	0	-	0.6	0	-

Table S2: H/D isotope effects on the substrate water exchange rates of the S_2 state of photosystem II core complexes isolated from wild-type (WT) and D1-D61A of *Synechocystis* sp. PCC 6803. The rate constants and fractions of PSII centers were obtained from global fits of the $^{16,18}O_2$ (m/z 34) and $^{18,18}O_2$ (m/z 36) data displayed as lines in **Figure S3.** The data were obtained at 10°C and pL 6.5. nd – not determined.

		**WT-PSII**		**D61A-PSII**			
S_2,		k_f	k_s	k_{f1}	k_{s1}	k_{f2}	k_{s2}
**H_2O**	**fraction, %**	100		85		15	
	**rate, s^{-1}**	84 ± 5	0.97 ± 0.03	> 300	15 ± 1	1.4 ± 0.9	0.4 ± 0.1
**D_2O (88%)**	**fraction, %**	100		76		24	
	**rate, s^{-1}**	76 ± 7	0.75 ± 0.03	> 300	8.7 ± 0.5	1.5 ± 0.6	0.16 ± 0.02
	**H/D effect**	1.1 ± 0.1	1.29 ± 0.07	nd	1.7 ± 0.2	~ 1	2.5 ± 0.7
	**corrected H/D**	1.3 ± 0.1	1.47 ± 0.08	nd	1.9 ± 0.2	nd	2.8 ± 0.8

Table S3: Simulations of the rates of isotopic equilibration between bulk water and the inner water pool surrounding the $Mn_4CaO_{5/6}$ cluster in photosystem II. Simulations were done as described in **SI Text 1** and illustrated in **Scheme 3** and **Figure S5**. **Simulation 1** reflects the situation that we propose for WT-PSII, while **Simulation 2** that of the D61A mutant. We have no constraints for setting the barrier height 1 in WT or mutant, it is thus taken from reference.[30] **Simulations 3-5** illustrate the effect of the inner water pool size on the exchange rates. **Simulation 6-8** show that variation of the number of water molecules in pools 1 and 2, or the variation of the exchange energy within these pools is inconsequential for the rate of isotopic equilibration of the inner pool (pool 3), as long as the exchange energies are 10.0 kcal mol^{-1} or lower. **Simulations 9-11** illustrate the effects of a single barrier on the exchange rates of a single water behind the barrier (compare to **Simulation 3**). **Simulations 12-16** probe several different variations in barrier heights as compared to **Simulation 1**.

**Simulation No.**	**No. of W in pool 1 (kcal mol^{-1})**	**Barrier 1, kcal mol^{-1}**	**No. of W in pool 2 (kcal mol^{-1})**	**Barrier 2, kcal mol^{-1}**	**No. of W in pool 3 (kcal mol^{-1})**	**Rate of W exchange in pool 3, s^{-1}**
1	**5 (2)**	**11.5**	**4 (5)**	**12.8**	**8 (2)**	89
2	5 (2)	11.5	4 (5)	**8.0**	8 (2)	690
3	5 (2)	11.5	4 (5)	12.8	**1**	640
4	5 (2)	11.5	4 (5)	12.8	**3** (2)	230
5	**3** (2)	11.5	4 (5)	12.8	**10** (2)	71
6	**2**(2)	11.5	**2**(5)	12.8	8 (2)	90
7	5 (2)	11.5	4 (**2**)	12.8	8 (2)	87
8	5 (**10**)	11.5	4 (**10**)	12.8	8 (**10**)	86
9	**1**	11.5	**1**	-	-	8100
10	**1**	**12.8**	**1**	**-**	**-**	800
11	**1**	**14.0**	**1**	**-**	**-**	80
12	5 (2)	**2.0**	4 (5)	12.8	8 (2)	90
13	5 (2)	**12.8**	4 (5)	**11.5**	8 (2)	60
14	5 (2)	**11.5**	4 (5)	**11.5**	8 (2)	450
15	5 (2)	**12.8**	4 (5)	**12.8**	8 (2)	40
16	5(2)	**15.0**	4(5)	**8.0**	8 (2)	1.3

SI References

1. J. Messinger, M. Badger and T. Wydrzynski, *Proc. Natl. Acad. Sci. USA*, 1995, **92**, 3209-3213.
2. W. Hillier, J. Messinger and T. Wydrzynski, *Biochemistry*, 1998, **37**, 16908-16914.
3. C. de Lichtenberg and J. Messinger, *Phys. Chem. Chem. Phys.*, 2020, **22**, 12894-12908.
4. W. Hillier and T. Wydrzynski, *Biochemistry*, 2000, **39**, 4399-4405.
5. G. Hendry and T. Wydrzynski, *Biochemistry*, 2003, **42**, 6209-6217.
6. G. Hendry and T. Wydrzynski, *Biochemistry*, 2002, **41**, 13328-13334.
7. J. Messinger, *Phys. Chem. Chem. Phys.*, 2004, **6**, 4764 - 4771.
8. H. Nilsson, T. Krupnik, J. Kargul and J. Messinger, *Biochim. Biophys. Acta*, 2014, **1837**, 1257-1262.
9. X. C. Li and P. E. M. Siegbahn, *Phys. Chem. Chem. Phys.*, 2015, **17**, 12168-12174.
10. L. Rapatskiy, N. Cox, A. Savitsky, W. M. Ames, J. Sander, M. M. Nowaczyk, M. Rögner, A. Boussac, F. Neese, J. Messinger and W. Lubitz, *J. Am. Chem. Soc.*, 2012, **134**, 16619-16634.
11. N. Cox and J. Messinger, *Biochim. Biophys. Acta*, 2013, **1827**, 1020-1030.
12. P. E. M. Siegbahn, *Acc. Chem. Res.*, 2009, **42**, 1871-1880.
13. P. E. M. Siegbahn, *J. Am. Chem. Soc.*, 2013, **135**, 9442-9449.
14. C. de Lichtenberg, A. P. Avramov, M. Zhang, F. Mamedov, R. L. Burnap and J. Messinger, *Biochim. Biophys. Acta*, 2021, **1862**, 148319.
15. P. E. M. Siegbahn, *Proc. Natl. Acad. Sci. USA*, 2017, **114**, 4966-4968.
16. B. B. Zhang and L. C. Sun, *Dalt. Trans.*, 2018, **47**, 14381-14387.
17. X.-C. Li, J. Li and P. E. M. Siegbahn, *J. Phys. Chem. A*, 2020, **124**, 8011-8018.
18. M. Ibrahim, T. Fransson, R. Chatterjee, M. H. Cheah, R. Hussein, L. Lassalle, K. D. Sutherlin, I. D. Young, F. D. Fuller, S. Gul, I. S. Kim, P. S. Simon, C. de Lichtenberg, P. Chernev, I. Bogacz, C. C. Pham, A. M. Orville, N. Saichek, T. Northen, A. Batyuk, S. Carbajo, R. Alonso-Mori, K. Tono, S. Owada, A. Bhowmick, R. Bolotovsky, D. Mendez, N. W. Moriarty, J. M. Holton, H. Dobbek, A. S. Brewster, P. D. Adams, N. K. Sauter, U. Bergmann, A. Zouni, J. Messinger, J. Kern, V. K. Yachandra and J. Yano, *Proc. Natl. Acad. Sci. USA*, 2020, **117**, 12624-12635.
19. J. Kern, R. Chatterjee, I. D. Young, F. D. Fuller, L. Lassalle, M. Ibrahim, S. Gul, T. Fransson, A. S. Brewster, R. Alonso-Mori, R. Hussein, M. Zhang, L. Douthit, C. de Lichtenberg, M. H. Cheah, D. Shevela, J. Wersig, I. Seuffert, D. Sokaras, E. Pastor, C. Weninger, T. Kroll, R. G. Sierra, P. Aller, A. Butryn, A. M. Orville, M. N. Liang, A. Batyuk, J. E. Koglin, S. Carbajo, S. Boutet, N. W. Moriarty, J. M. Holton, H. Dobbek, P. D. Adams, U. Bergmann, N. K. Sauter, A. Zouni, J. Messinger, J. Yano and V. K. Yachandra, *Nature*, 2018, **563**, 421-425.
20. P. E. M. Siegbahn, *Chem. Eur. J.*, 2006, **12**, 9217-9227.
21. V. L. Pecoraro, M. J. Baldwin, M. T. Caudle, W.-Y. Hsieh and N. A. Law, *Pure Appl. Chem.*, 1998, **70**, 925-929.
22. J. P. McEvoy and G. W. Brudvig, *Phys. Chem. Chem. Phys.*, 2004, **6**, 4754-4763.
23. K. N. Ferreira, T. M. Iverson, K. Maghlaoui, J. Barber and S. Iwata, *Science*, 2004, **303**, 1831-1838.
24. M. Kusunoki, *Biochim. Biophys. Acta*, 2007, **1767**, 484-492.
25. G. Renger, *Biochim. Biophys. Acta*, 2012, **1817**, 1164-1176.
26. T. A. Corry and P. J. O'Malley, *J. Phys. Chem. Lett.*, 2020, **11**, 4221-4225.
27. W. Hillier and J. Messinger, in *Photosystem II. The Light-Driven Water:Plastoquinone Oxidoredutase*, eds. T. Wydrzynski and K. Satoh, Springer, Dordrecht, 2005, vol. 22, pp. 567-608.
28. J. P. McEvoy and G. W. Brudvig, *Chem. Rev.*, 2006, **106**, 4455-4483.
29. D. A. Pantazis, *ACS Catal.*, 2018, **8**, 9477-9507.
30. S. Vassiliev, T. Zaraiskaya and D. Bruce, *Biochim. Biophys. Acta*, 2012, **1817**, 1671-1678.
